# Comparative complete chloroplast genome analysis of *Cucurbita* (*Cucurbitaceae*) species revealed insights into phylogenetic evolution, adaptive pressure, and lineage diversification

**DOI:** 10.3389/fpls.2026.1803791

**Published:** 2026-03-27

**Authors:** Xinbi Jia, Putao Wang, Cong Zhou, Sikandar Amanullah, Fangning Wu, Chenghong Zeng, Pin Zhang, Qianglong Zhu

**Affiliations:** 1 Jiangxi Province Key Laboratory of Vegetable Cultivation and Utilization, Jiangxi Agricultural University, Nanchang, Jiangxi, China; 2 Key Laboratory of Dong Medical Research of Hunan Province, Biomedical Research Institute, Hunan University of Medicine, Huaihua, Hunan, China; 3 Engineering Research Center for Horticultural Crop Germplasm Creation and New Variety Breeding, College of Horticulture, Hunan Agricultural University, Changsha, China; 4 Department of Horticultural Science, North Carolina State University, Mountain Horticultural Crops Research and Extension Center, Mills River, NC, United States

**Keywords:** codon usage bias, complete chloroplast genomes, *Cucurbita*, DNA barcoding, genome organization, phylogenetic analysis, selective pressure

## Abstract

*Cucurbita* is an economically and nutritionally important genus within the Cucurbitaceae. However, its taxonomy and evolutionary relationships remain difficult to resolve due to extensive phenotypic diversity, domestication history, and frequent hybridization. In this study, we generated ten complete chloroplast genomes (CPGs), including newly sequenced genomes of *Cucurbita moschata* and *C. maxima* and *de novo* assemblies of eight additional species. We conducted a comprehensive comparative phylogenomic analysis across twelve *Cucurbita* species by integrating the two previously published genomes. The obtained results showed that the CPGs of these *Cucurbita* species were 157,309 bp ± 568 bp in size and encoded 130~131 annotated genes, including 86 protein-coding genes, 36~37 transfer RNA (tRNA) genes, and 8 ribosomal RNA (rRNA) genes. All these CPGs presented a conserved quadripartite circular structure, containing an LSC, an SSC and two IR regions. Analysis of codon usage bias indicated leucine (Leu) was the most abundant amino acid, and the relative synonymous codon usage (RSCU) values of thirty codons were greater than 1.00. A total of nine simple sequence repeat (SSR) primers were validated as highly polymorphic among *Cucurbita* species, and the *trnL*-*trnF* region was identified as the most suitable DNA barcode in *Cucurbita*. The chloroplast phylogenomic analysis of 61 representative Cucurbitaceae species confirmed *Cucurbita* as a strongly supported monophyletic core group, with *C. cordata* and *C. digitata* in a basal position. Furthermore, *Cucurbita* was found to be sister to a group of Benincaseae containing *Citrullus*, *Zehneria*, and *Cucumis*. A total of seven genes (*atpE*, *atpF*, *matK*, *ndh*A, *rpoC2*, *ycf1*, and *ycf2*) harbored significant selective sites under positive selection and promoted the plant development and environmental adaptation of *Cucurbita*. This study substantially enriches chloroplast genomic resources for *Cucurbita* and provides an integrated framework for resolving its phylogenetic relationships, evolutionary history, taxonomic classification, and adaptive diversification, with important implications for taxonomy, breeding, and conservation of cucurbit germplasm.

## 1 Introduction

The *Cucurbita* genus is part of the Cucurbitaceae family and comprises approximately 12–15 species (Sanjur et al., 2002; Schaefer and Renner, 2011; Paris, 2016). Most *Cucurbita* species originated in the Americas and are important cultivated crops with significant economic and medicinal value (Sun et al., 2017). Fruit and seed extracts of *Cucurbita* species exhibit pharmacological activities, including antidiabetic, hepatoprotective, antioxidant, anticancer, and anti-inflammatory effects (Roman-Ramos et al., 2012; Abdelkader et al., 2022; Al-Shaheen et al., 2013; Marbun et al., 2018). *C. moschata*, *C. maxima*, and *C. pepo* are the three key cultivated species of *Cucurbita* in economic terms (Sanjur et al., 2002; Sun et al., 2017; Zhu et al., 2021). They are rich in nutritional compounds and possess great medicinal components (Nyabera et al., 2021), such as carotenoids, vitamins, polysaccharides, dietary fibers, and microelements, which are obtained mainly from ripened fruits, foliage, petals, and seeds. Furthermore, *C. argyrosperma* is valued for its edible seeds, which serve as a suitable model for studying *Cucurbita* domestication (Barrera-Redondo et al., 2021), and *C. ficifolia* is widely used as a rootstock to enhance stress tolerance in Cucurbitaceae (Zhou et al., 2021).

The wild species (*C. cordata*, *C. digitata*, *C. foetidissima*, and *C. pedatifolia*) are a group of important germplasm resources with high drought tolerance and disease resistance (Khoury et al., 2020), and their seeds and roots are considered prime sources for the extraction of starch, oil, and secondary metabolites (Berry et al., 1975; DeVeaux and Shultz, 1985). *Cucurbita* shows greater fruit morphological diversity compared with other groups in the *Cucurbita*ceae family (Paris, 2016), driven by long-standing domestication, cultivation, breeding, and germplasm exchange among global regions. The cultivar diversity of *Cucurbita* has increased remarkably, with many cultivars showing both unique and shared morphological traits, such as broadly ovate foliage, hollow creeping stems, monoecious inflorescences, inferior ovaries, yellow corollas, spherical or ellipsoidal fruits, and flat seeds, etc. These similarities not only complicate the identification and classification of *Cucurbita* but also hinder the analysis of their phylogenetic relationships.

As the specialized organelle of photosynthesis, the chloroplastthe supplies the energy necessary for plant growth and is vital for developmental processes (Kuroda and Maliga, 2003). Moreover, genes in complete CPGs have been reported to be important for phenotypic traits in *Cucurbita*, and pericarp pigment and cotyledon senescence are regulated by the differential expression of genes in the CPG (Schaffer et al., 1984; Mishev et al., 2011). The CPGs of most plants contain about 130 genes, including tRNA, rRNA, and protein-coding genes (Daniell et al., 2016). Plant CPGs have a moderate rate of nucleotide evolution, smaller genome size, and unique maternal inheritance compared with mitochondrial or nuclear genomes (Zhang et al., 2017; Zhou et al., 2023), and they are regarded as important molecular markers for taxonomy according to the Consortium for the Barcode of Life (Hollingsworth, 2011).

The first-ever CPGs of *C. ficifolia* and *C. pepo* were reported in our earlier research (Zhang et al., 2018b; Zhou et al., 2021), and the CPG of *C. argyrosperma* subsp. *argyrosperma* has also been reported in other peer-reviewed research publications (Barrera-Redondo et al., 2019). However, there is a lack of directed research reports on the CPGs of *C. moschata* and *C. maxima*, while their sequences are available in the NCBI nucleotide database (*C. moschata*: OQ442842.1, NC_036506.1; *C. maxima*: NC_036505.1, OK129338.1). Only chloroplast scaffolds or partial sequences from these and other *Cucurbita* (*C. argyrosperma*, *C. cordata*, *C. digitata*, *C. ecuadorensi*s, *C. foetidissima*, *C. lundelliana*, *C. okeechobeensis*, and *C. pedatifolia*) have been used in phylogenetic and domestication studies (Wilson et al., 1992; Schaefer et al., 2009; Zheng et al., 2013; Kistler et al., 2015). Numerous archaeological findings demonstrate that *C. maxima* and *C. moschata* emerged independently in southern and northern regions of South America and experienced high levels of genetic divergence in their secondary domestication centers of China-Japan and India-Myanmar (Ferriol and Pico, 2008; Sun et al., 2017). Moreover, the spread of cultivated *Cucurbita* was accompanied by seed exchange and hybridization, leading to notable genomic structural variation in some regional populations (Sun et al., 2017; He et al., 2024). Therefore, expanding sampling from diverse geographic sources and enriching the complete CPG resources of *Cucurbita* will facilitate a more comprehensive understanding of phylogenetic relationships and genetic diversity in this genus.

In this study, the CPGs of Chinese *C. moschata* and *C. maxima* were sequenced and assembled, and the CPGs of an additional eight *Cucurbita* species were *de novo* assembled based on whole-genome sequencing data from the Sequence Read Archive (SRA) database of the National Center for Biotechnology Information (NCBI) website. We also conducted a comparative analysis of the 12 *Cucurbita* CPGs together with the published CPGs of *C. pepo* and *C. ficifolia* from our prior studies, including genome organization, codon usage bias, repeat sequences, divergence hotspots, adaptive evolution, and phylogenetic relationships. The primary objectives were as follows: (1) to present ten assembled CPGs of *Cucurbita* species; (2) to uncover the chloroplast genome organization, develop divergence hotspots and, develop SSRs as molecular markers for species taxonomy in *Cucurbita*; and (3) to analyze their phylogenetic relationships and coding genes during adaptive evolution in *Cucurbita*.

## 2 Materials and methods

### 2.1 Research materials and DNA extraction

The five most cultivated species (*C. moschata*, *C. maxima*, *C. argyrosperma*, *C. pepo*, and *C. ficifolia* in *Cucurbita*) were selected as research material and cultivated at the horticultural teaching station of Jiangxi Agricultural University, Nanchang, China. The young, disease-free leaves were harvested from seedlings after one month, at the four-leaf stage. Following rapid freezing in liquid nitrogen, the samples were preserved in -80 °C until required for sequencing. Total genomic DNA was extracted from these leaf tissues with a DNA extraction kit (M5; Mei5 Biotechnology Co., Ltd., China). Subsequently, the integrity and concentration of the purified DNA were assessed by spectrophotometry (Nanodrop 2000) and agarose gel electrophoresis (1%, w/v). High-quality genomic DNA was then used for subsequent genome sequencing and SSR polymorphism analysis.

### 2.2 Chloroplast genome sequencing, *de novo* assembly, and genome annotation

The paired-end (PE) libraries with an average insert length of 500 bp were prepared from the purified DNA of *C. moschata* and *C. maxima.* Genomic sequencing was performed on an Illumina HiSeq 2500 platform (BGI, Tianjin, China) using 2 × 150 bp paired-end reads. Subsequent filtering of poor-quality reads was performed with Trimmomatic, resulting in 1–2 Gb of high-quality sequence data per sample following quality trimming, with an adequate average read coverage across the CPGs of *C. moschata* (1021×) and *C. maxima* (825×). To further obtain and improve the CPGs of *Cucurbita*, the clean sequencing data of *C. argyrosperma* (SRR7685402), *C. cordata* (SRR2531290), *C. digitata* (SRR26763386), *C. ecuadorensis* (SRR26753257), *C. foetidissima* (SRR11573110), *C. lundelliana* (SRR26763385), *C. okeechobeensis* (SRR26761657), and *C. pedatifolia* (SRR26761658) were downloaded from the SRA data of the NCBI website according to their accession number, respectively.

The high-quality PE sequenced reads were used for *de novo* assembly and annotation of the chloroplast genome via the following steps. First, these clean PE reads were *de novo* assembled using Plasmidspades.py in SPAdes (version 3.12.0) with the default k-mer size (-k 21,33.55,77,99,127) (Bankevich et al., 2012). Second, the scaffolds belonging to the chloroplast genome were retrieved via BlastN (version 2.7.1) (https://ftp.ncbi.nlm.nih.gov/blast/executables/LATEST/) against the CPG of *C. ficifolia* (NC_058583.1) and manually sorted and concatenated into a long sequence representing the draft chloroplast genome. Third, the gaps in the draft chloroplast genome were closed using GapCloser (version 1.12-r6) (Luo et al., 2012), and filtered PE reads were finally mapped to the gap-free draft chloroplast genome to confirm the completion of the chloroplast genome. The CPG was annotated using CPGAVAS2 (http://www.herbalgenomics.org/cpgavas2) (Shi et al., 2019) and GeSeq (https://chlorobox.mpimp-golm.mpg.de/geseq.html) (Tillich et al., 2017), and the problems in the annotation were manually corrected using the open-source software Integrative Genomics Viewer (IGV, version 2.6.3) (Robinson et al., 2011). A circular CPG map was created using OGDRAW-Draw Organelle Genome Maps (https://chlorobox.mpimp-golm.mpg.de/OGDraw.html) (Greiner et al., 2019). These newly assembled CPGs were submitted to the China National GenBank Database (CNGBdb) (https://db.cngb.org/) and the online dataset of the NCBI website (https://www.ncbi.nlm.nih.gov/#!/home/contact).

### 2.3 Codon usage bias analysis

The RSCU was determined with CodonW (version 1.4.2) to assess codon usage bias (Jia et al., 2025). The greater the RSCU value (>1.00), the more it suggested that the use of a codon has codon usage bias, and if the RSCU value equals 1.00 or <1.00, it suggested that the use of a codon has no codon usage bias or a lower frequency than expected, respectively (Sharp and Li, 1986). A heatmap for cluster analysis of the RSCU values of the CPGs of 12 *Cucurbita* species was constructed using an upgraded TBtools software (version 1.108) (Chen et al., 2020).

### 2.4 Repeated sequence identification and polymorphic SSR validation

Dispersed repeats (DRs), palindromic repeats (PRs), tandem repeats (TRs), and simple single repeats (SSRs) were four important repetitive sequences that were analyzed in the 12 *Cucurbita* CPGs. The position and size of the DRs and PRs were identified via the local implementation of large scale bioinformatics software “Vmatch” (version 2.3.1) (Kurtz, 2010), with a minimum repeat sequence length of 30 bp and a similarity threshold of at least 90% for the two repeats. The overlapping repeats were then manually filtered. We used the Tandem Repeats Finder online tool (https://tandem.bu.edu/trf/trf.html) for TRs identification, applying alignment parameters of 2 (match), 7 (mismatch), and 7 (indel), and a minimum TR length of 7 bp (Benson, 1999). MISA-web (https://webblast.ipk-gatersleben.de/misa/) was used to detect SSRs, with the following minimum repeat parameters applied: 10 for mononucleotide, 5 for dinucleotide, and 4 for tri-, tetra-, penta-, and hexanucleotide repeats (Beier et al., 2017).

To develop polymorphic SSR markers, the aligned genome sequences of 12 *Cucurbita* species were used to screen and predict polymorphic SSRs. The primers flanking the polymorphic SSRs were designed using Primer 3 (version 0.4.0) (https://primer3.ut.ee/) (Untergasser et al., 2012), and these primers were oligo-synthesized by Beijing Qingke Biotechnology Co., Ltd. The genomic DNA samples of five representative available species (*C. moschata*, *C. maxima*, *C. argyrosperma*, *C. ficifolia*, and *C. pepo*) were used to validate the polymorphisms of these SSR primers. The polymerase chain reaction (PCR) analysis system was used as described in our previous study (Cao et al., 2021).

### 2.5 Whole chloroplast genomes comparison

To explore the variations in the features and CPGs structures of the *Cucurbit*a, the CPGs of *C. pepo* (NC_038229.1) and *C. ficifolia* (NC_058583.1) were downloaded from the NCBI according to their accession numbers. The CPG of *C. moschata* was used as a reference for comparison and visualization via the mVISTA program (https://genome.lbl.gov/vista/mvista/submit.shtml) with the default settings (Frazer et al., 2004). The IRs region borders of 12 *Cucurbita* species were analyzed using the online program CPJSdraw (https://github.com/xul962464/CPJSdraw) (Li et al., 2023). Genome collinearity and gene rearrangement were analyzed using the Mauve (Darling et al., 2004).

### 2.6 Nucleotide variability and adaptive evolution analysis

To analyze the interspecific variations in *Cucurbita* CPGs, nucleotide variability (Pi) in the 12 *Cucurbita* species was detected using a user-friendly Windows software package DnaSP (version 6.12.03), with 600 bp windows and a step size of 200 bp (Rozas et al., 2017). We further assessed intraspecific variation within *C. moschata* and *C. maxima* using the same software and parameters. The clustering analysis was performed on MEGA (version 11.0) based on the neighbor-joining (NJ) method (Kumar et al., 2018).

To explore the adaptive evolution in *Cucurbita* protein-coding genes, the nonsynonymous-to-synonymous substitution rate ratios (Ka/Ks) were calculated with the EasyCodeML software (version 1.41). Analyses were performed under the preset running mode of the software, which provides built-in parameters and standardized pipelines for selection detection. Four pairs of nested site models (M0 vs. M3, M1a vs. M2a, M7 vs. M8, and M7a vs. M8a) were used, and significance was determined by likelihood ratio tests (LRT) based on the raw p-values directly output by the software with a threshold of p < 0.05, following the standard usage protocol of EasyCodeML (Gao et al., 2019).

### 2.7 Phylogenetic analysis and divergence time estimation

For the construction of phylogenetic association, CPGs of 61 Cucurbitaceae species were selected, with *Corynocarpus aevigata* (Corynocarpaceae) as the outgroup (**Supplementary Table 1**). The total sequences were aligned using MAFFT software (version 7.129) (Katoh et al., 2019). The maximum likelihood (ML) tree was constructed using the MEGA (version 11.0) with the maximum composite likelihood (MLC) method (Kumar et al., 2018). ML tree analysis was performed with IQ-TREE 2 (Minh et al., 2020), while Bayesian inference (BI) was conducted using MrBayes (version 3.2.7) under the Markov chain Monte Carlo (MCMC) framework, with two separate runs of 20 million generations each and trees were sampled every 1,000 generations (Ronquist et al., 2012).

Prior to tree construction, the optimal nucleotide substitution model was chosen with ModelTest-NG (version 0.2.0, Darriba et al., 2020) based on the Akaike information criterion (AIC) (Posada and Buckley, 2004). Branch support was assessed based on bootstrap values (BP) for ML and posterior probabilities (PP) for BI. Phylogenetic relationships were reconstructed using four distinct datasets in response to the variation in molecular evolutionary rates across the different CPG regions: (1) complete CPG sequences, (2) the large single-copy (LSC) region, (3) the small single-copy (SSC) region, and (4) one inverted repeat (IRa) region. All phylogenetic trees were plotted using ChiPlot (https://www.chiplot.online/) (Xie et al., 2023).

To estimate the divergence time of *Cucurbita* species, the molecular clock in MEGA software was used to analyze the divergence time (Tamura et al., 2021). The ML tree was taken as the original tree, and the time tree was computed using 3.04–3.84 mya as calibration constraints according to a previous study (Sun et al., 2017).

## 3 Results

### 3.1 Analysis of genome organization of *Cucurbita* CPGs

**Figure 1:**
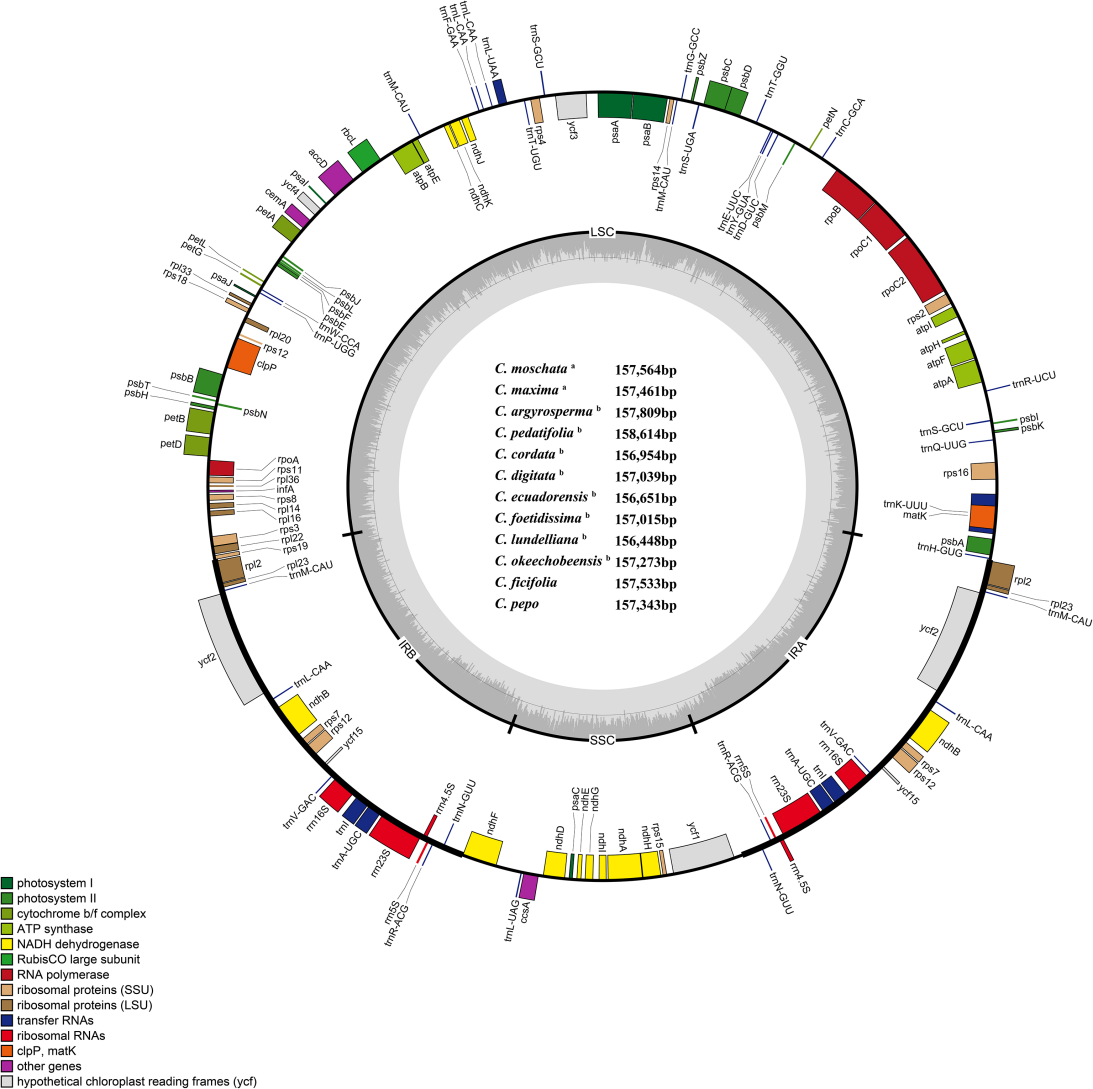
Gene map of the CPGs of *Cucurbita*. Superscript ^a^indicates the CPGs sequenced and assembled in this study; superscript ^b^denotes the CPGs assembled using existing databases. Genes are transcribed clockwise in the inner circle and counterclockwise in the outer circle. Genes in different functional groups are color-coded. The grey of the inner circle represents the percentage of GCs in the CPG, and the light grey represents 1-GC%.

A total of 10 complete CPGs of *Cucurbita* species, including *C. moschata*, *C. maxima*, *C. argyrosperma*, *C. cordata*, *C. digitata*, *C. ecuadorensis*, *C. foetidissima*, *C. lundelliana*, *C. okeechobeensis*, and *C. pedatifolia*, were *de novo* assembled based on their clean DNA sequence data (**Figure 1**, **Supplementary Figure 1**, **Table 1**). These 10 complete CPGs were subsequently submitted to GenBank or CNGBdb, and accession numbers were obtained, as detailed in **Table 1**. The CPGs of *C. pepo* and *C. ficifolia* were downloaded from the NCBI website. The CPGs of the 12 *Cucurbita* species presented a conserved quadripartite organization, including one large single-copy (LSC), one small single-copy (SSC), and two inverted repeat (IR) regions. The lengths of the 12 CPGs ranged from 156,448 bp (*C. lundelliana*) to 158,614 bp (*C. pedatifolia*), with an average and standard deviation of 157,309 bp ± 568 bp. The average lengths of the LSC and SSC, and IR regions were 87,809 bp, 18,120 bp and 25,689 bp; however, the IR regions presented the highest coefficient of variation, 0.012, respectively. The average GC content was 37.1%, whereas GC content varied among different regions. A substantially higher GC content was observed in the IR regions (42.7-43.1%) relative to both the LSC (34.8-34.9%) and SSC (31.2-31.5%) regions.

**Table 1:** Comparison of CPG organization among 12 *Cucurbita* species.

Species	*C. moschata*	*C. maxima*	*C. argyrosperma*	*C. ficifolia*	*C. pepo*	*C. pedatifolia*
Size (bp	157,564	157,461	157,809	157,533	157,343	158,614
LSC (bp)	88,182	88,046	88,387	88,113	87,971	87,322
SSC (bp)	18,157	18,171	18,182	18,144	18,168	18,128
IR (bp)	25,613	25,622	25,620	25,638	25,602	26,582
Overall GC content (%)	37.1	37.1	37.2	37.1	37.1	37.1
GC content in LSC (%)	34.9	34.9	34.8	34.8	34.9	34.8
GC content in SSC (%)	31.5	31.4	31.2	31.5	31.4	31.5
GC content in IR (%)	43	43	42.9	43	43	42.7
Total Genes Number	131	131	131	131	131	131
CDS Number	86	86	86	86	86	86
tRNAs Number	37	37	37	37	37	37
rRNAs Number	8	8	8	8	8	8
Duplicated genes	18	18	18	18	18	18
Accession number of chloroplast genome in GenBank/CNGBdb	N_002031915	N_002031914	NC_065148.1/ OL782153.1	NC_058583.1/MZ578000.1	NC_038229.1/MH031787.1	NC_065199.1/ON597625.1
Species	*C. cordata*	*C. digitata*	*C. ecuadorensis*	*C. foetidissima*	*C. lundelliana*	*C. okeechobeensis*
Size (bp	156,954	157,039	156,651	157,015	156,448	157,273
LSC (bp)	87,939	88,062	87,137	87,554	87,020	87,982
SSC (bp)	18,141	18,119	18,002	17,997	18,050	18,181
IR (bp)	25,437	25,429	25,756	25,732	25,689	25,555
Overall GC content (%)	37.1	37.1	37.1	37.1	37.1	37.1
GC content in LSC (%)	34.8	34.8	34.8	34.8	34.8	34.9
GC content in SSC (%)	31.2	31.2	31.5	31.4	31.4	31.4
GC content in IR (%)	43	43.1	42.9	42.9	42.9	43
Total Genes Number	131	131	131	131	131	131
CDS Number	86	86	86	86	86	86
tRNAs Number	37	37	37	37	36	37
rRNAs Number	8	8	8	8	8	8
Duplicated genes	18	18	18	18	18	18
Accession number of chloroplast genome in GenBank/CNGBdb	N_001486273	N_001486272	N_001486274	N_001486275	N_001486271	NC_065149.1/ OL782154.1

The number of annotated genes identified in the 12 CPGs ranged from 130-131, including 86 protein-coding genes with different gene functions, 36–37 tRNA genes, and 8 rRNA genes (**Table 2**). Among these, 18 genes were found to be duplicated at two IR regions, including seven protein-coding genes (*rpl23*, *ndhB*, *rps12*, *rpl2*, *rps7*, *ycf1*, and *ycf2*), seven tRNA genes (*trnA*-*UGC*, *trnL*-*CAA*, *trnI*-*GAU*, *trnN*-*GUU*, *trnI*-*CAU*, *trnV*-*GAC*, and *trnR*-*ACG*), and four rRNA genes (*rrn4.5*, *rrn5*, *rrn16*, and *rrn23*).

**Table 2:** List of annotated genes in the chloroplast genomes of the 12 *Cucurbita* species.

Gene functions	Gene categories	Names of genes
Photosynthesis	Subunits of ATP synthase	*atpA*, *atpB*, *atpE*, *atpF*^b^, *atpH*, *atpI*
	Subunits of NADH dehydrogenase	*ndhA*^b^, *ndhB*^ab^, *ndhC*, *ndhD*, *ndhE*, *ndhF*, *ndhG*, *ndhH*, *ndhI*, *ndhJ*, *ndhK*
	Subunits of cytochrome	*petA*, *petB*^b^, *petD*^b^, *petL*, *petN*, *petG*
	Subunits of photosystem I	*psaA*, *psaB*, *psaC*, *psaI*, *psaJ*
	Subunits of photosystem II	*psbA*, *psbB*, *psbC*, *psbD*, *psbE*, *psbF*, *psbH*, *psbI*, *psbJ*, *psbK*, *psbL*, *psbM*, *psbN*, *psbT*, *psbZ*
	Subunit of rubisco	*rbcL*
Self-replication	Large subunit of ribosome	*rpl2*^ab^, *rpl14*, *rpl16*^b^, *rpl20*, *rpl22*, *rpl23*^a^, *rpl32*, *rpl33*, *rpl36*
	DNA-dependent RNA polymerase	*rpoA*,*rpoB*,*rpoC1*^b^,*rpoC2*
	Small subunit of ribosome	*rps2*, *rps3*, *rps4*, *rps7^a^*, *rps8*, *rps11*, *rps12^ab^*, *rps14*, *rps15*, *rps16*^b^, *rps18*, *rps19*
	rRNA Genes	*rrn4.5^a^*, *rrn5^a^*, *rrn16^a^*, *rrn23^ab^*
	tRNA Genes	*trnH-GUG*, *trnK-UUU*^b^, *trnQ-UUG*, *trnS-GCU*, *trnG-UCC*^b^, *trnR-UCU*, *trnC-GCA*, *trnD-GUC*, *trnY-GUA*, *trnE-UUC*, *trnT-GGU*, *trnS-UGA*, *trnG-GCC*, *trnfM-CAU*, *trnS-GGA*, *trnT-UGU*, *trnL-UAA*^b^, *trnF-GAA*, *trnV-UAC*^b^, *trnM-CAU*, *trnW-CCA*, *trnP-UGG*, *trnI-CAU*, *trnL-CAA*, *trnV-GAC*, *trnI-GAU*^b^, *trnA-UGC^b^, trnR-ACG*, *trnN-GUU^a^*, *trnL-UAG*, *trnR-ACG*, *trnA-UGC*^b^, *trnI-GAU*^b^, *trnV-GAC*^b^, *trnL-CAA*, *trnI-CAU*
Other functions	Subunit of AcetylCoA-carboxylase	*accD*
	c-type cytochrome synthesis gene	*ccsA*
	Envelope membrane protein	*cemA*
	Protease	*clpP^c^*
	Maturase	*matK*
	Translation initiation factor	*infA*
unknown function	Conserved open reading frames	*ycf1*^a^, *ycf2*^a^, *ycf3*^c^, *ycf4*

^a^indicates duplicated gene in IRs; ^b^indicates a gene that contains one intron; ^c^indicates a gene that contains two introns.

### 3.2 Codon usage bias

**Figure 2:**
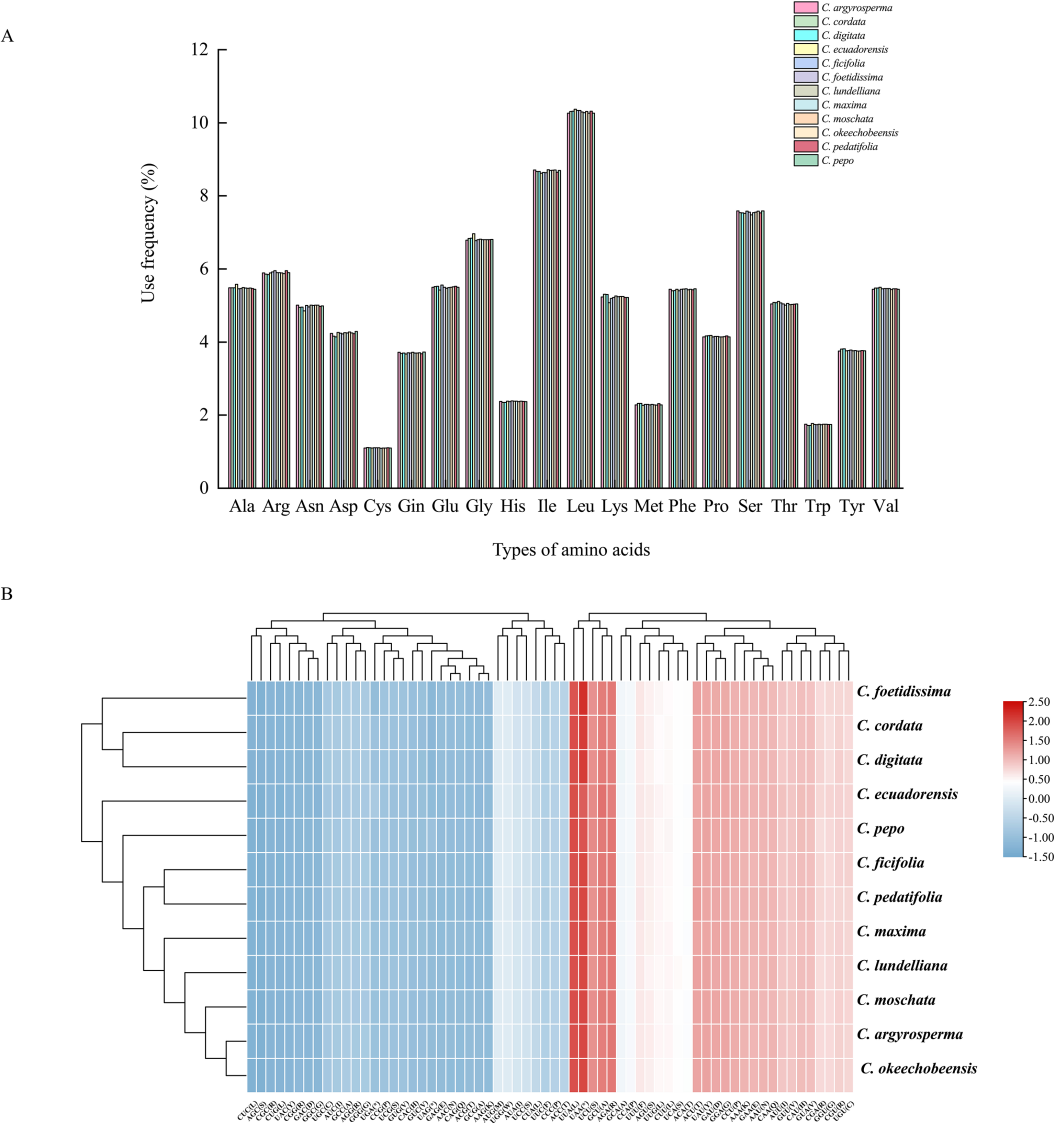
Analysis of codon usage bias of 12 CPGs in *Cucurbita*. **(A)** Amino acids and stop codon proportions in the 12 CPGs of *Cucurbita*. The X-axis represents the types of amino acids; the Y-axis represents the frequency of amino acids in the 12 CPGs of *Cucurbita*. **(B)** Heatmap analysis for the RSCU of *Cucurbita* chloroplast genes.

A total of 79 protein-coding genes were detected as common in the CPGs of 12 *Cucurbita* species and included codons from 20,302 (*C. digitata*) to 20,496 (*C. pepo*). The frequencies of amino acid and codon usage in these protein-coding genes were then analyzed. Among the 20 amino acids, leucine (Leu) was the most prevalent, with a frequency of 10.2–10.37%, followed by isoleucine (Ile), with a frequency of 8.62–8.71%, whereas cysteine (Cys) was the rarest, with a frequency of only 1.09–1.11% (**Figure 2A**). Further analysis of the RSCU values revealed that thirty codons with values above 1.00, indicating that their usage was more frequent than anticipated. Apart from Leu-UUG, the third position codons of all codons (RSCU > 0.1) were A/U, such as the top five biased codons (Stop (*)-UAA, Leu-UUA, Ser-UCU, Arg-AGA, and Ala-GCU), with high RSCU values (>1.68) (**Figure 2B**). Methionine (Met) and tryptophan (Trp) lacked codon usage bias and had RSCU values of 1.00. Thirty-two codons showed RSCU values less than 1.00.

### 3.3 Analysis of identified repeats and developed SSRs

**Figure 3:**
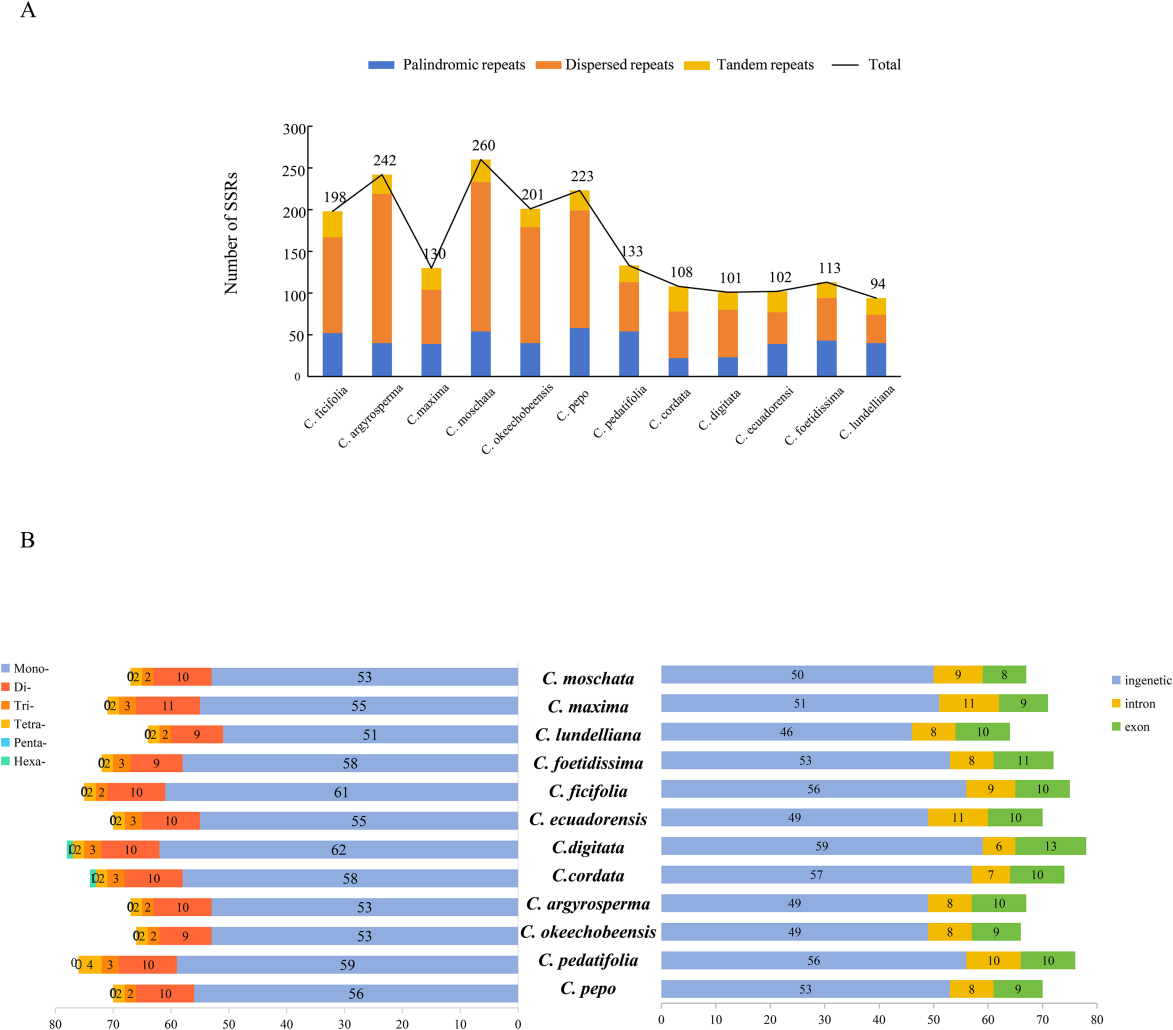
Types and existence of repeated sequences and SSRs in 12 CPGs of *Cucurbita*. **(A)** Number of three repeat types in 12 CPGs. **(B)** Number of SSR types in 12 CPGs.

Tandem, dispersed, and palindromic repeats were detected across the 12 CPGs of *Cucurbita*, as represented in **Figure 3A**. Total repeats ranged from 94 (*C. lundelliana*) to 260 (*C. moschata*), with an average of 159. These CPGs contained the greatest number of dispersed repeats (average of 92), followed by palindromic repeats (41), and the lowest number of tandem repeats (21). Additionally, we identified six types of SSRs among the 12 *Cucurbita* species. The total number of SSRs ranged from 64 (*C. lundelliana*) to 78 (*C. digitata*), with an average of 71 (**Figure 3B**). Mononucleotide repeats showed the highest abundance (78.8%), compared with dinucleotide repeats at 14.1% and trinucleotide repeats at 4.2%. The majority of SSR loci were located within intergenic regions (52/71, 73.2%), and only a few SSRs were in the coding region (9/71, 12.7%).

**Figure 4:**
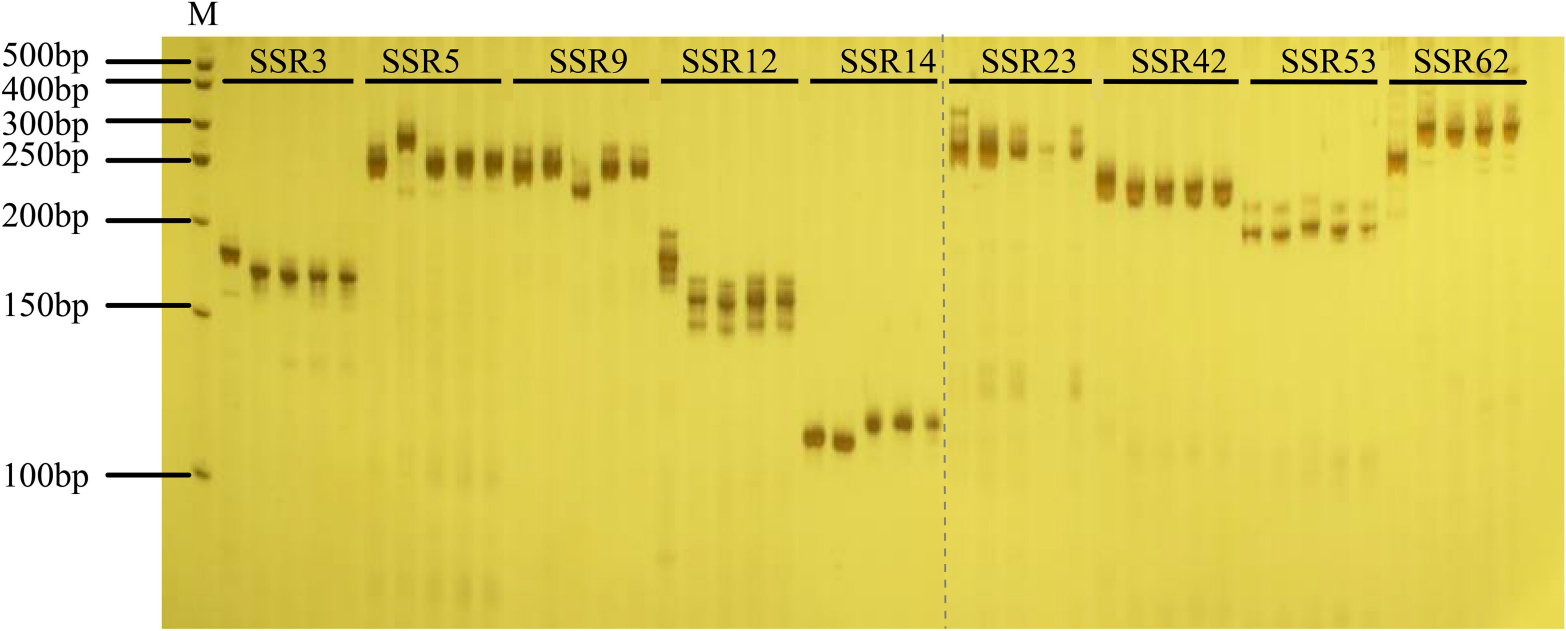
Amplification results of nine SSR polymorphic primers in the five *Cucurbita* cultivars. The nine polymorphic SSR primers used were SSR3, SSR5, SSR9, SSR12, SSR14, SSR23, SSR42, SSR53, and SSR62, and the corresponding bands of each pair of primers were *C. ficifolia*, *C. pepo*, *C. moschata*, *C. argyrosperma*, and *C. maxima*. M represents the 500 bp marker. The figure has been cropped in the gray lined area, and 50bp marker has been cropped out of the figure.

The percentage of all repeats in the CPG ranged from 2.95% (*C. lundelliana*) to 12.45% (*C. argyrosperma*), with an average of 7.01% (**Supplementary Table 2**). A total of 42 SSR loci with polymorphisms were identified after sequence alignment analysis of 12 CPGs of *Cucurbita* (**Supplementary Table 3**). Subsequently, 42 pairs of SSR primers were developed based on the sequences around these SSR loci (**Supplementary Table 4**). PCR amplification was conducted using DNA samples extracted from five distinct *Cucurbita* cultivars as templates. Electrophoresis analysis showed that nine SSR primers produced clear polymorphic bands among the five *Cucurbita* species (**Figure 4**).

### 3.4 IR/SC boundary and genome rearrangement

**Figure 5:**
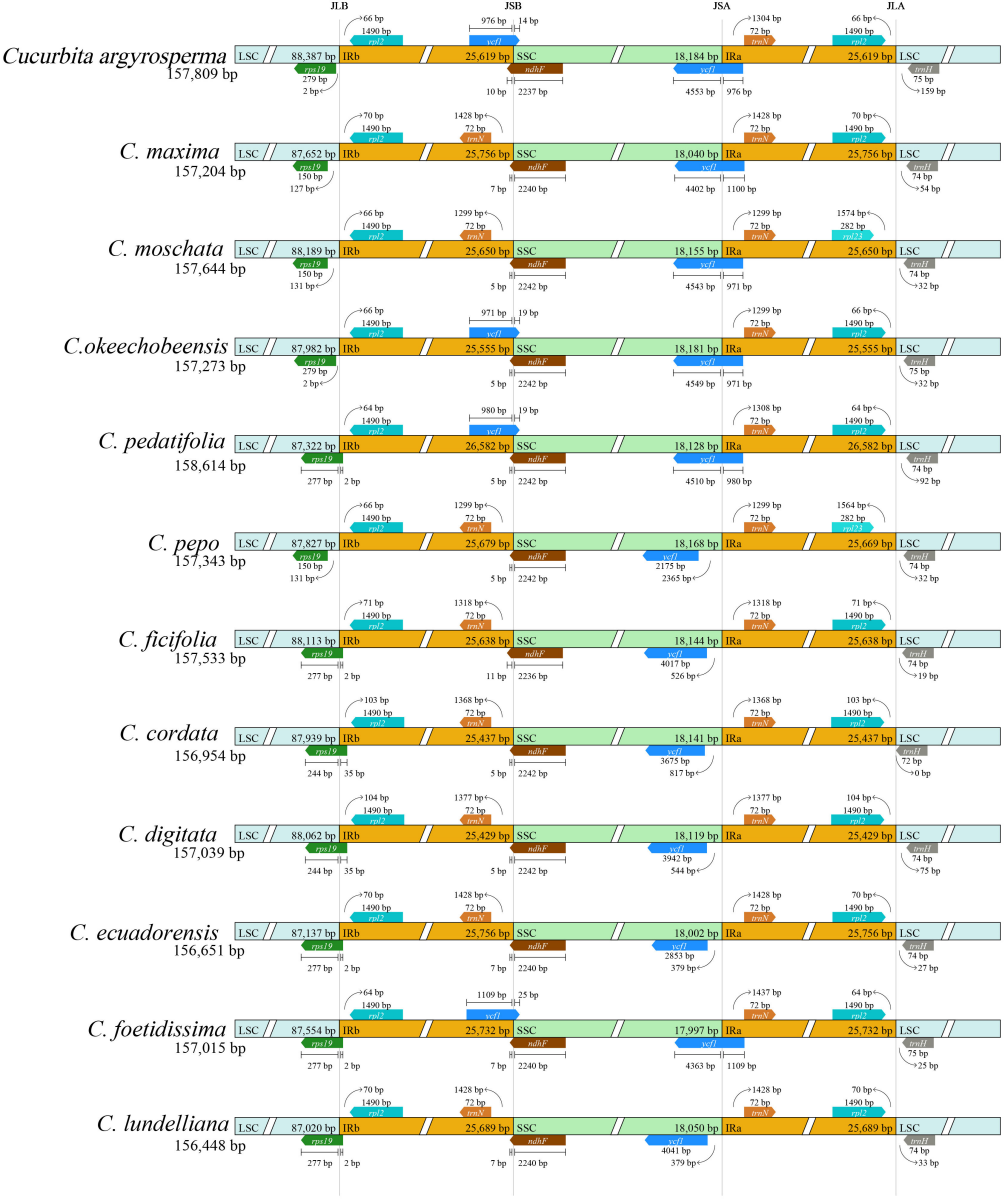
Comparison of the IR boundaries of the CPGs of 12 *Cucurbita* species. The genes on the positive chain are represented by the tracks representing each accession, whereas the genes on the negative chain are represented by the tracks representing each accession. The number on the arrow indicates the distance between the gene start or end position and the joint point of the boundary. The abbreviations in the figure indicate junction sites in the CPG: JLA (IRa/LSC), JLB (IRb/LSC), JSA (SSC/IRa) and JSB (IRb/SSC). Genes are drawn in different color boxes. The figure does not represent the sequence length to scale.

A detailed genome comparison was conducted to assess the variations in the IR/SC boundaries of the 12 *Cucurbita* species. The results revealed that the JLA (IRa/LSC) boundary of the *Cucurbita* showed greater variation than the other IR/SC boundaries (**Figure 5**). These *Cucurbita* species can be divided into three groups according to the position of *rps19* at JLA: Group I contains *C. argyrosperma*, *C. moschata*, *C. maxima*, *C. okeechobeensis*, and *C. pepo*; Group II contains *C. ficifolia*, *C. pedatifolia*, *C. ecuadorensis*, *C. foetidissima*, and *C. lundelliana*; Group III only contains *C. cordata* and *C. digitata*. The *rps19* gene was near to JLA in Group I, whereas the *rps19* gene in Group II overlapped JLA by 2 bp, and the Group III had a longer overlap length (35 bp). Collinearity analysis of the 12 CPGs revealed an absence of genomic rearrangements (**Supplementary Figure 2**), suggesting highly conserved genome structure among *Cucurbita* species.

### 3.5 Analysis of divergence in complete chloroplast genome and hotspot regions

**Figure 6:**
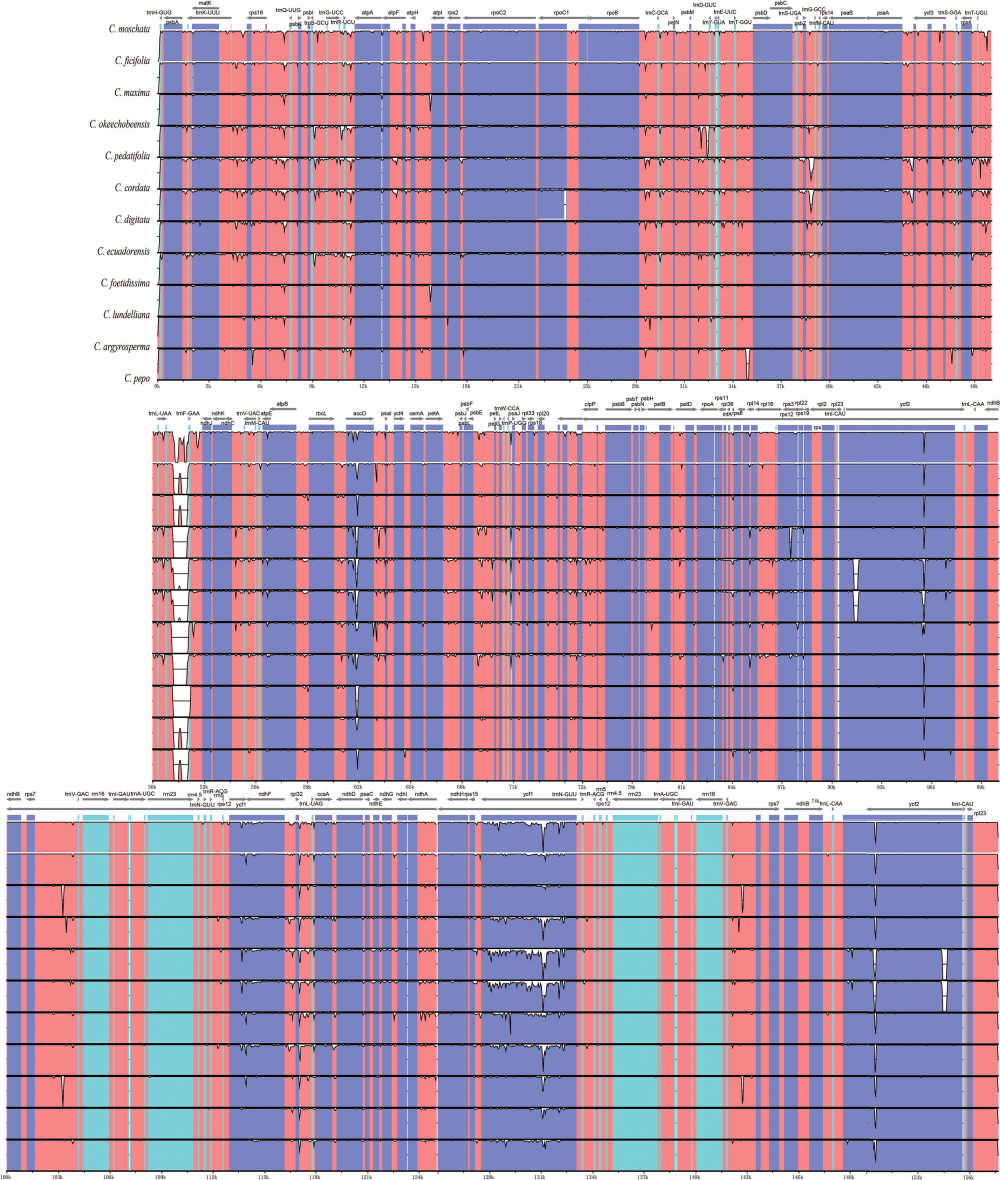
Visualization alignment of the CPGs of 12 *Cucurbita* species.

**Figure 7:**
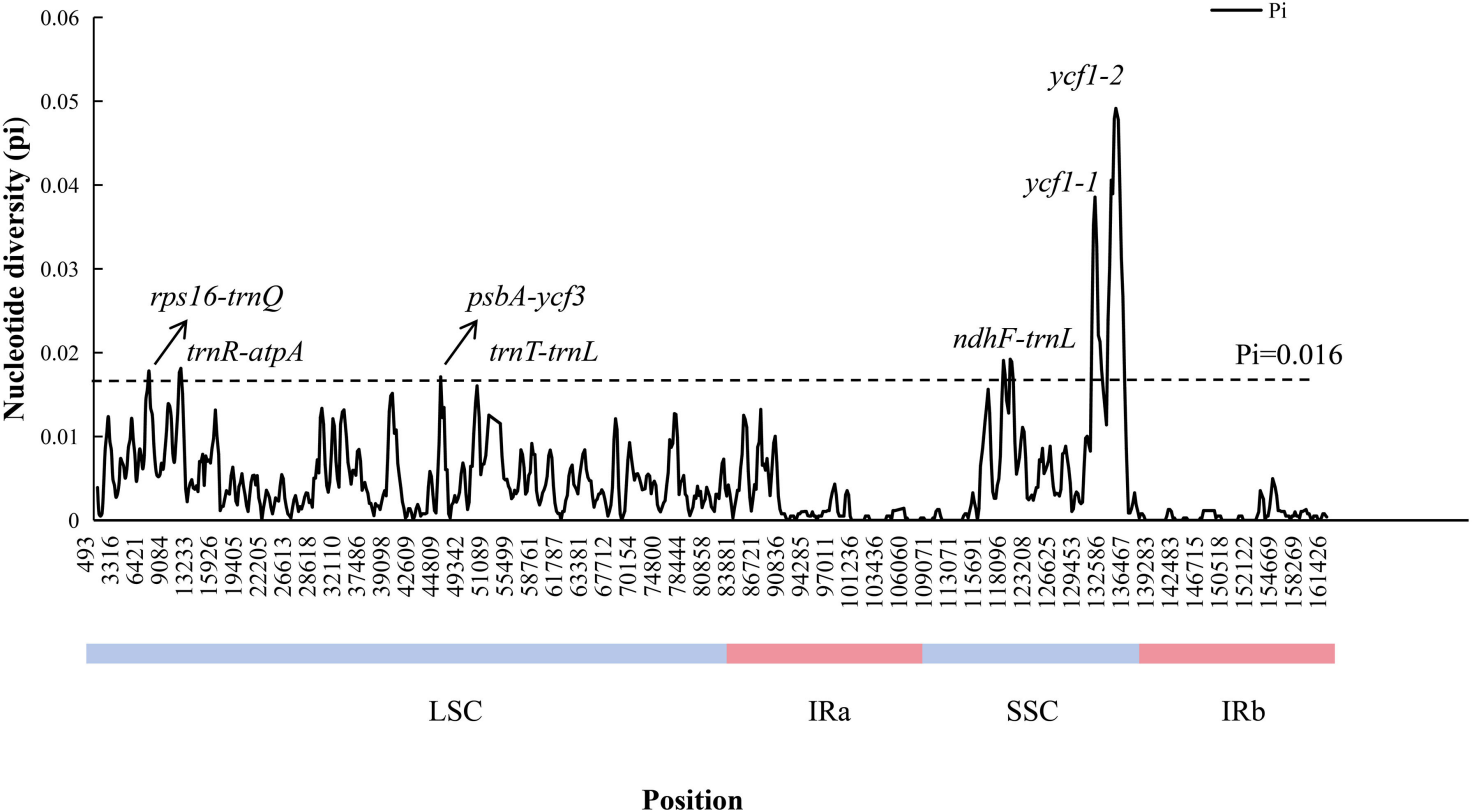
Midpoint position sliding window map of nucleotide diversity (Pi) in the CPGs of 12 *Cucurbita* species. Window length: 600 bp; step size: 200 bp.

To assess interspecific variation among the 12 *Cucurbita* species, whole CPGs were aligned using MAFFT, and comparative analysis was conducted with mVISTA and DnaSP. The alignment revealed greater sequence variation in SC regions than in IR regions, with non-coding region showing higher variability than coding regions. Substantial gaps were identified in the *trnL*-*trnF* region of *Cucurbita* species, and the *trnF* gene was absent in *C. lundelliana*. Among coding regions, the *ycf1* gene displayed the highest sequence variation (**Figure 6**). Nucleotide diversity (Pi) analysis further revealed that the SSC region as the most variable, followed by the LSC region, while the two IR regions showed the lowest variation (**Figure 7**). Seven highly divergent regions (Pi ≥ 0.016) were identified as follows: *ycf1*-*1* (0.049), *ycf1*-*2* (0.039), and *ndhF*-*trnL* (0.019) in the SSC region, and *rps16*-*trnQ* (0.018), *trnR*-*atpA* (0.018), *psaA*-*ycf3* (0.017), and *trnL*-*trnF* (0.016) in the LSC region, all of which were rich in variable sites (VSs), parsimony informative sites (Pins), average K-2P distances, and discrimination success rates (DSRs) (**Table 3**). Notably, the *trnL*−*trnF* region showed 100% DSR and suitable aligned length, with clear discrimination of the 12 *Cucurbita* species in the NJ clustering tree (**Supplementary Figure 3**), suggesting its potential as a promising DNA barcode for *Cucurbita*.

**Table 3:** The variability of the seven divergent regions in *Cucurbita*.

Hotspot regions	Location^a^	Aligned length	Pi	VS	Pin	Average K-2P distance	DSRb
*rps16*-*trnQ*	6366-7774	1445	0.018	63	43	0.013	83%
*trnR-atpA*	10625-11678	1063	0.018	50	29	0.016	83%
*psaA-ycf3*	44594-45605	1054	0.017	40	31	0.013	58%
*trnL-trnF*	48868-50129	1326	0.016	47	30	0.011	100%
*ndhF-trnL*	115961-117992	2106	0.019	115	65	0.016	83%
*ycf1-1*	127630-129693	2106	0.039	158	107	0.023	100%
*ycf1-2*	129694-132401	2882	0.049	287	193	0.032	83%

a The location is confirmed based on the CPG of *C. moschata*. VS, Variable sites; Pin, Parsimony informative sites; DSR, Discrimination success rate (%) based on distance method.

Intraspecific variation was then investigated separately for *C. moschata* and *C. maxima*. For *C. moschata*, the three CPGs of *C. moschata* displayed multiple nucleotide variations, including base substitutions, insertions, and deletions (**Supplementary Figure 4A**). The SSC region showed particularly high variability, with Pi ≥ 0.4. For *C. maxima*, 20 mutation sites with a dispersed distribution were identified, including two highly polymorphic loci (TC/CG/TC and A/C/A, A/A/C, and */A/G, respectively) (**Supplementary Figure 4B**). The clustering analysis further showed genetic divergence for both N_802031915 and N_802031914 from their respective conspecific individuals (**Supplementary Figure 4C**).

### 3.6 Phylogenetic analysis and divergence time estimation

**Figure 8:**
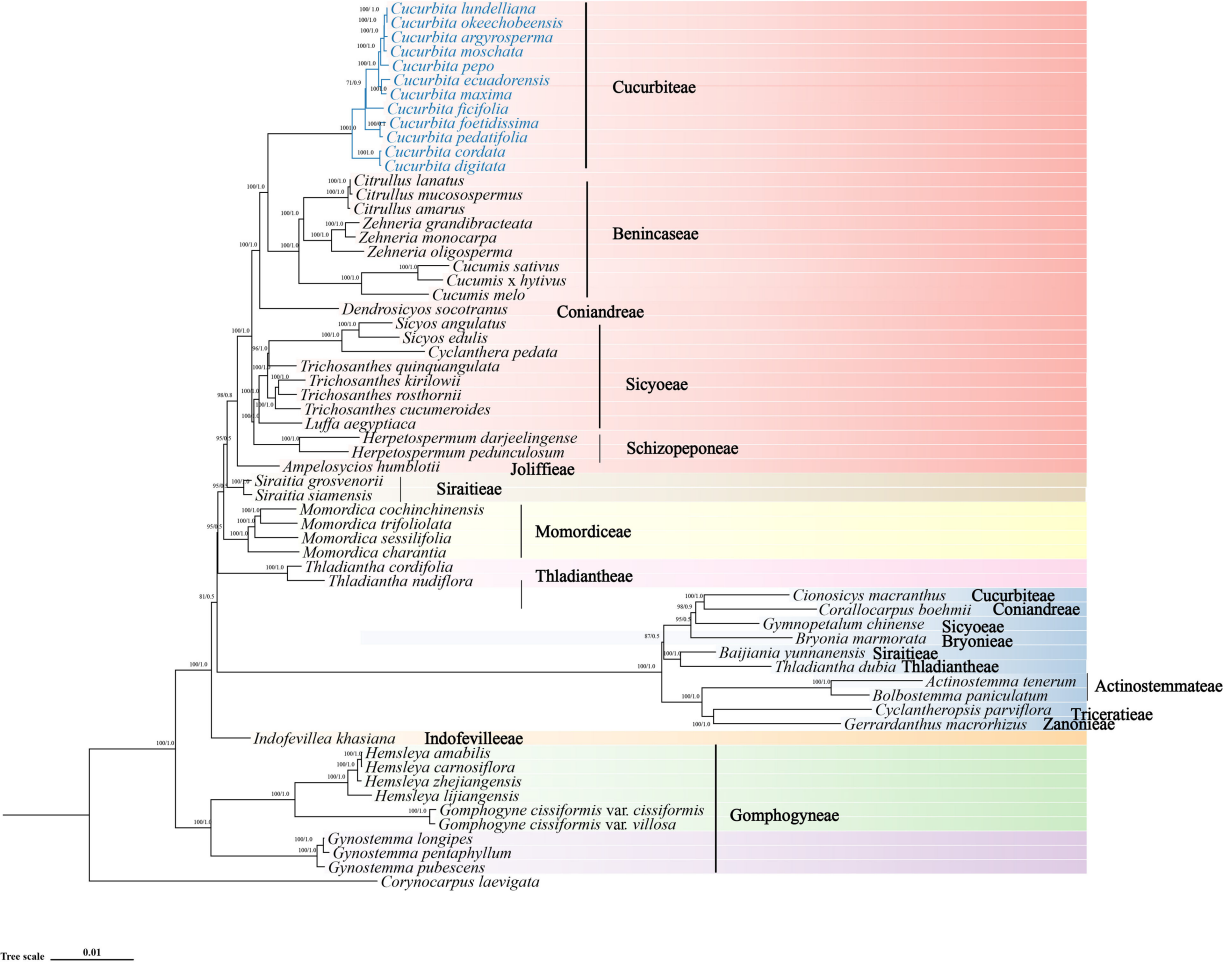
The phylogenetic relationships among the 61 Cucurbitaceae species were analyzed using both ML and BI methods. Support values for all nodes as followed: ML bootstrap values (1-100)/BI posterior probabilities (0-1.0). *Corynocarpus aevigata* (Corynocarpaceae) was used as the outgroup. Different clades are marked with distinct color blocks, and 12 *Cucurbita* species are highlighted in blue font.

The phylogenetic trees were constructed using CPGs of 61 representative Cucurbitaceae species, representing 15 tribes in the family. A total of four datasets were employed to build both ML and BI trees (**Figure 8**, **Supplementary Figure 5**), and the best-fit models were TVM+F+R6 for the ML tree and TVM+I+G4 for the BI tree based on complete CPGs. It was shown that the genetic distance within tribes was significantly smaller than that between tribes. The monophyletic nature of most families was verified by high support values (100/1.0). These 61 Cucurbitaceae species were classified into eight distinct clades based on the topological structure, each highlighted with different color modules. The Gomphogyneae lineage (including *Hemsleya*, *Gynostemma*, and *Gomphogyne*) diverged earlier than all other tribes in the Cucurbitaceae.

**Figure 9:**
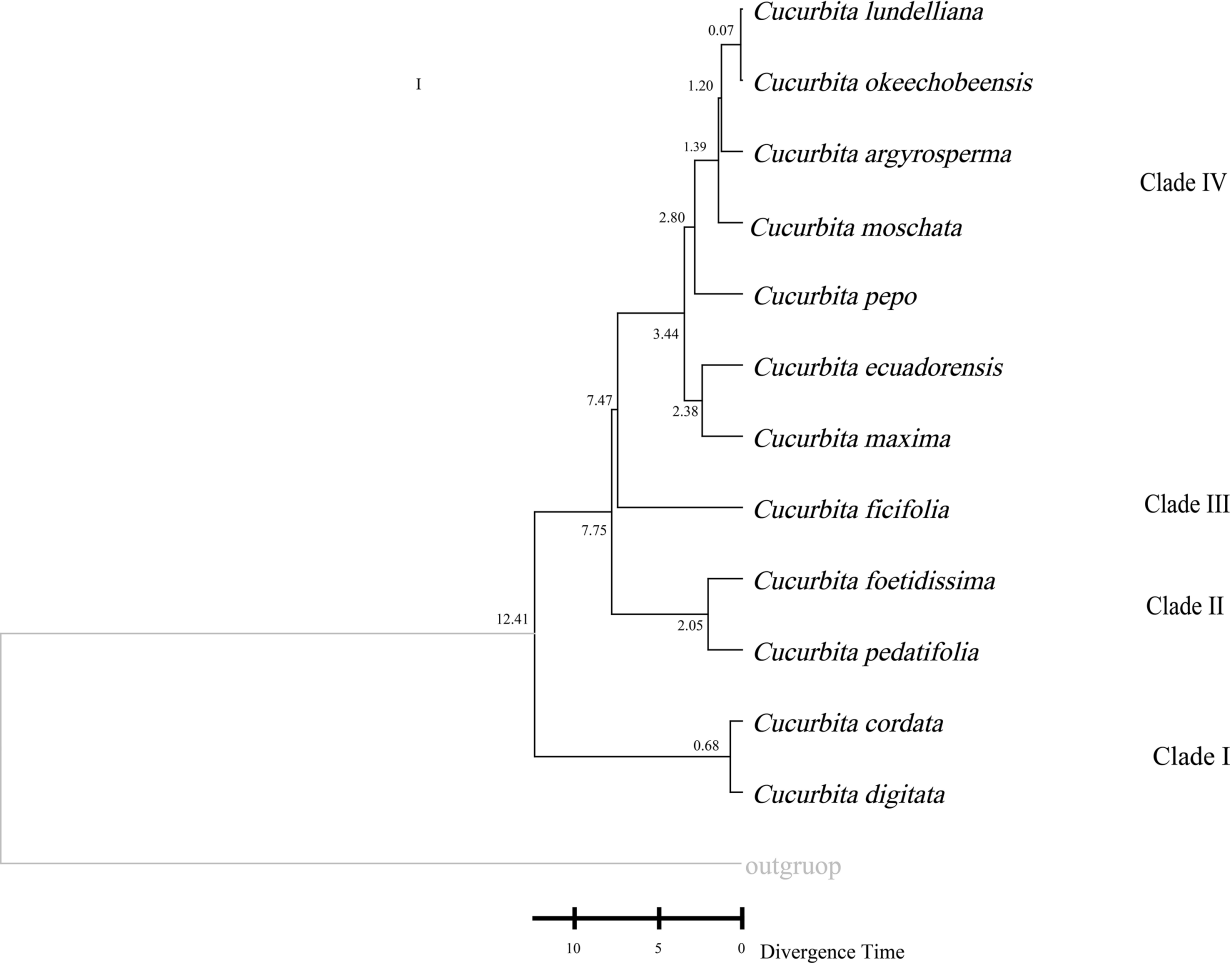
Divergence time estimation of *Cucurbita* based on CPGs.

*Cucurbita* is a key genus within the Cucurbitaceae family. Species in this genus showed a clear clustering pattern: *C. maxima*, *C. ecuadorensis*, *C. pepo*, *C. moschata*, *C. lundelliana*, *C. argyrosperma*, and *C. okeechobeensis* grouped into a subclade, while the wild species *C. cordata* and *C. digitata* constituted the basal lineage of *Cucurbita*. However, consistent results were obtained across the other three datasets, and *Cionosicys macranthus* did not cluster with the core *Cucurbita* clade and showed clear genetic divergence from the main *Cucurbita* lineage, while it grouped with the tribe in trees constructed from the LSC and IRa datasets. Furthermore, divergence time estimation for *Cucurbita* based on the ML tree showed that Clades I and II would start evolving independently at approximately 12.41 and 7.75 mya, that Clades III and IV would separate and begin evolving independently around 7.47 mya, and that three important cultivars (*C. pepo*, *C. maxima*, and *C. moschata*) in Clade IV would start evolving independently at 2.38 mya, 2.80 mya, and 1.39 mya, respectively (**Figure 9**).

### 3.7 Gene selective pressure analysis

For the estimation of gene selection pressure, the ω ratio was calculated for the 79 common protein-coding genes in the 12 *Cucurbita* CPGs (**Table 4**). In the M2a model, *rpoC2* showed the highest ω2 value (128.99), while *matK* had the lowest (9.93). Notably, *ycf1* possessed the most significant positive selection signal, with the lowest LPR p-value (0.5797E−5) and six positively selected sites, indicating that the *ycf1* gene was under stronger selective pressure than the other CPG genes in *Cucurbita*. Moreover, *atpF* and *rpoC2* each presented three positive sites each, and *atpE*, *matK*, *ycf2*, and *ndhA* each presented one positive site.

**Table 4:** The results of positive selective pressure analysis in M2a, M7 vs. M8 model.

Gene name	Model	np	LnL	ω2(M2a)	LPR p-value	Positive site
*atpE*	M8	26	-478.61	17.10	0.2066	82 K 0.958*
	M7	24	-480.19			
*atpF*	M8	26	-1782.04	25.06	0.0022	235 E 0.992**, 239 F 0.952*, 311 E 0.958*
	M7	24	-1788.18			
*matK*	M8	26	-1943.85	9.93	0.2268	21 P 0.966*
	M7	24	-1945.33			
*ndhA*	M8	26	-2864.26	56.15	0.0040	296 P 0.988*
	M7	24	-2869.80			
*rpoC2*	M8	26	-5523.40	128.99	0.0011	37 W 0.996**; 778 S 0.971*; 961 Y 0.971*
	M7	24	-5530.24			
*ycf1*	M8	26	-1436.85	18.80	0.5797E-5	21 I 0.997**, 22 K 0.991**, 16 Y 0.992**, 179 C 0.991**, 220 T 0.991**, 248 G 0.991**
	M7	24	-1448.91			
*ycf2*	M8	26	-8988.80	26.77	0.0638	2099 C 0.980*
	M7	24	-8991.55			

*: means P < 0.05; **: means P < 0.01.

## 4 Discussion

### 4.1 Genome organization and codon patterns

*Cucurbita* is one of the most agriculturally and economically vital genera in the Cucurbitaceae family (Renner and Schaefer, 2016). However, the available complete CPG resources for *Cucurbita* remain relatively limited. The scaffold-form chloroplast genome sequence of *Cucurbita* reported by Kistler et al. (2015) contains 2–6 sequence gaps and only 101–104 annotated genes, far fewer than the typical gene count of complete CPGs (**Supplementary Table 5**). In the present study, the CPGs of ten *Cucurbita* species were obtained. As with the previously published CPGs of *C. pepo* and *C. ficifolia*, all genomes were processed using a unified bioinformatics pipeline to ensure consistent assembly. This approach minimized technical bias and provided a highly reliable foundation for comparative genomics. A comprehensive whole-chloroplast genome comparison analysis was conducted across these 12 *Cucurbita* species. All experimental procedures were carried out following the aforementioned methods. The CPG lengths of the 12 *Cucurbita* species ranged from 156,448 bp (*C. lundelliana*) to 158,614 bp (*C. pedatifolia*), with an average length of 157,309 bp and a standard deviation of 568 bp. The variation in chloroplast genome length among *Cucurbita* species was greater than that reported in *Citrullus* and *Gynostemma* (Zhou et al., 2023; Zhang et al., 2017). This suggested a higher level of interspecific difference within *Cucurbita* in the Cucurbitaceae family. The CPG length was determined by SCs (LSC and SSC) and IRs (IRa and IRb), and the variable coefficient of the IRs’ length was greater than that of the SCs region of *Cucurbita* in this study, indicating that the contraction and expansion of IRs promoted the variation in the CPG length of *Cucurbita*.

Consistent with reports in other Cucurbitaceae species, the *Cucurbita* chloroplast genome was found to contain 86 protein-coding, eight rRNA, and 36–37 tRNA genes (Xia et al., 2023; Xue et al., 2019). Leu and Ile in the protein-coding genes were the most abundant amino acid, and thirty codons had the RSCU > 1.00, suggesting that a strong bias in amino acid and codon usage was present in the CPG of *Cucurbita*, further supporting that the codon usage bias is a general characteristic of plant chloroplast genomes (Yang et al., 2023; Liu et al., 2024). The exploration of codon patterns would provide insight into the evolutionary studies of species and theoretical research on genetic modifications of CPGs (Jiang et al., 2023).

### 4.2 Detection of repeats and SSR marker development

Repeated sequences in CPGs could serve a crucial function in generating variation and species selection and domestication (Guo et al., 2007; Li et al., 2022). In this study, by combining the analysis results of repeat sequence patterns and phylogenetic clades, we found that while Clades I and II had significantly lower totals of TRs, PRs, and DRs. They harbored a greater abundance of SSRs compared to Clades III and IV. This finding suggests that an increase in TRs, PRs, and DRs, along with a decrease in SSRs during evolution could promote the selection and domestication of *Cucurbita*. All these repeats constituted approximately 7% of the whole CPG. This proportion was lower than the percentage of repeats (approximately 40%) in the nuclear genome of *Cucurbita* (Montero-Pau et al., 2018). Furthermore, nine SSRs were found to be significant polymorphisms among the five *Cucurbita* species in our study. These results suggest that chloroplast-based SSRs have potential application value in species identification and evolutionary analysis (Jayaswall et al., 2023; Zhu et al., 2023).

### 4.3 Variation in high variation regions

Highly variable regions in the CPG could serve as molecular markers or DNA barcodes applications in species discrimination, taxonomic classification, and phylogeny reconstruction (Schaefer et al., 2009; Song et al., 2017; Feng et al., 2024). Despite the absence of genomic rearrangements in the CPG of *Cucurbita* species verified through IR/SC boundary and collinearity analyses in our study, these CPGs presented large interspecific sequence divergence. The seven high-Pi regions (*ycf1-1*, *ycf1-2*, *ndhF-trnL*, *rps16-trnQ*, *trnR-atpA*, *psaA-ycf3*, and *trnL-trnF*) are suitable candidates for DNA barcoding to study the phylogeny and genetic diversity of *Cucurbita* species. Among them, *trnL-trnF* region is especially promising due to its discrimination success rates and the aligned length in *Cucurbita*, and it has been successfully applied as a specific molecular marker in taxonomic and domestication research on Cucurbitaceae species (Sebastian et al., 2010). *Ycf1* is a 214-kDa protein of the Tic complex and has the second-longest open reading frame in the CPG (Dong et al., 2015). The most variable coding regions in the CPG of *Cucurbita* was observed within the *ycf1* gene (*ycf1–1* and *ycf1-2*). This variability has also been noted in the genera *Zingiber* (Jiang et al., 2023), *Celastrus* (Liu et al., 2024), and *Cicer* (Temel et al., 2022), indicating its potential as a viable DNA barcode (Dong et al., 2015). Moreover, five other highly variable regions (*ndhF-trnL*, *rps16-trnQ*, *trnR-atpA*, *psaA-ycf3*, *and trnL-trnF*) were in the noncoding regions, suggesting that the noncoding regions could constitute the greatest resource for molecular marker development, as supported by related studies (Song et al., 2017).

To date, only two CPGs each from *C. moschata* (OQ442842.1, NC_036506.1) and *C. maxima* (NC_036505.1, OK129338.1) were available in the NCBI. By incorporating our newly sequenced and assembled genomes in this study, we assessed intraspecific variation within these key cultivated species. Our analysis revealed that the SSC region was a mutation hotspot in *C. moschata*, whereas 20 dispersed polymorphic loci were identified in *C. maxima*. Furthermore, clustering patterns further showed distinct intraspecific lineages for both species, suggesting notable genetic divergence. Similar findings have been reported in investigations on the CPGs of 160 C*. ficifolia* individuals from 31 populations in Yunnan, confirming that these landraces possess high genetic diversity to adapt to local climatic conditions (He et al., 2024). These findings demonstrate that intraspecific variation in chloroplast genome structure and base composition is common in *Cucurbita*.

### 4.4 Phylogenetic relationship and divergence

Chloroplast phylogenomic analysis of 61 Cucurbitaceae species, based on both the complete genome and its structural partitions (LSC, SSC, IR), was conducted to establish a robust evolutionary framework for clarifying the relationships and diversification in *Cucurbita*. The stability of phylogenetic signals could be effectively verified by comparing the topological consistency across these four datasets (Zhang et al., 2018a). *Cucurbita* was strongly supported as monophyletic in all analyses, whereas *Cionosicys macranthus* clustered with *Cucurbita* only in trees derived from the LSC and IR regions. This pattern suggests that *C. macranthus* and *Cucurbita* share compatible core functional gene sequences in the LSC region and have a highly conserved IR region. *C. cordata* and *C. digitata* formed a relatively basal position in the *Cucurbita* lineage, indicating that they diverged early in the evolution of *Cucurbita*.

The estimation of divergence time revealed that they began independent evolution approximately 12.41 million years ago (mya) and likely retain more chloroplast genome characteristics of the ancestral *Cucurbita* species. The times at which three important cultivars (*C. pepo*, *C. maxima*, and *C. moschata*) started evolving independently were estimated at 2.38 mya, 2.80 mya, and 1.39 mya, respectively. This long evolutionary history has generated abundant genetic diversity in *Cucurbita*, which provides a valuable genetic resource for ensuring food security and expanding disease resistance (Grumet et al., 2021; Fenn et al., 2026).The results further support the traditional classification of *Cucurbita* based on fossils, key traits, limited molecular markers, and chloroplast sequences (Kistler et al., 2015; Castellanos-Morales et al., 2018; Zheng et al., 2013). Therefore, it is necessary to enrich the CPG data of additional *Cucurbita* species to clarify their phylogenetic relationships and provide a more reliable theoretical basis for the conservation and agricultural utilization of its genetic resources.

### 4.5 Adaptive evolution of chloroplast genes in *Cucurbita*

Analysis of selection pressures on chloroplast protein-coding genes provides valuable insights into the adaptive evolution of *Cucurbita* species. Seven genes (*atpE*, *atpF*, *matK*, *ndhA*, *rpoC2*, *ycf1*, and *ycf2*) harbored significant selection sites under positive selection based on ω2 values. *Ycf1* had the most significantly positive selective sites, followed by *rpoC2* and *atpF*. The *Ycf1* (Tic214) is the first chloroplast-encoded protein that directly participates in chloroplast protein import. As the largest subunit of the chloroplast inner envelope membrane translocon complex (TIC), *ycf1* acts as a core scaffold during the translocation of nuclear-encoded proteins across the chloroplast double membrane, and is essential for maintaining the semi-autonomous nature of chloroplasts (Dong et al., 2015; Xing et al., 2025); *ycf2* is an another plastid-encoded protein that acts as the largest motor component of the chloroplast protein translocon complex, originating from the plastid-encoded *ftsH* gene while retaining an essential ATPase domain (Kikuchi et al., 2018; Xing et al., 2025); *rpoC2* gene encodes the β subunit of plastid‐encoded RNA polymerase (PEP), which forms the core PEP complex together with other subunits such as *rpoA* and *rpoB* (Shimad et al., 1990). By participating in the assembly of the PEP complex, *rpoC2* directly regulates the transcription efficiency of chloroplast‐related genes, thereby affecting photosynthesis and energy metabolism in plants. In *Clivia miniata* var. *variegata*, a deletion mutation in the *rpoC2* gene downregulates the transcription of 28 chloroplast genes, impairs chloroplast biogenesis and thylakoid membrane development, and ultimately results in a yellow-striped leaf phenotype (Wu et al., 2023). The *atpF* gene, along with *atpE* and *ndhA*, is under positive selection and plays key roles in carbon fixation and photosynthesis (Daniell et al., 2008; Radomski et al., 2013; Huang et al., 2019). In the rice *ylws* mutant, splicing defects in *atpF*, *ndhA*, and other genes lead to significantly reduced transcription, impaired PEP activity, and abnormal chloroplast development, resulting in an albino phenotype (Lan et al., 2023). The *matK* gene encodes an essential enzyme known as intron maturase K, which is vital for the processing of Group II transcriptional introns in RNA (Hertel et al., 2013). Therefore, the changes in these chloroplast functional genes under selective pressure could promote the development and environmental adaptability of *Cucurbita*.

## 5 Conclusion

This study expanded the genomic resources for *Cucurbita* by providing ten new chloroplast genomes. The comprehensive comparative analyses indicated that *Cucurbita* species were more conserved in genomic organization, codon usage bias, and genome reorganization; however, differences were observed in SSRs and SC/IR boundaries. In the CPG of 12 *Cucurbita* species, thirty codons displayed RSCU values above 1.00, and Leu was the most abundant amino acid. A total of nine SSR primers were developed and showed strong polymorphism among *Cucurbita* species. However, based on the results of sequence variability and clustering analysis, the *trnL*-*trnF* region could serve as the optimal DNA barcode for the rapid identification of *Cucurbita*. Phylogenetic analysis confirmed the close phylogenetic relationship between *Cucurbita* and the tribe Benincaseae (including *Citrullus*, *Zehneria*, and *Cucumis*). *C. cordata* and *C. digitata* were the earliest diverging lineages in *Cucurbita*, having diverged from other members of *Cucurbita* around 12.41 mya. Furthermore, a total of 7 genes (*atpE*, *atpF*, *matK*, *ndhA*, *rpoC2*, *ycf1*, and *ycf2*) were found to possess significant sites of positive selection and are believed to be critical for promoting plant development and environmental adaptation in *Cucurbita*. In addition, the evaluated genetic diversity in *C. moschata* and *C. maxima* with multiple variation sites showed significant changes in each species. We believe that our study provides a basis for understanding the genetic differences in the chloroplast genome of *Cucurbita* and the domestication of cultivars.

## Data Availability

The data presented in this study is publicly available as follows. The raw sequencing data of C. moschata and C. maxima were deposited in the CNGBdb (https://db.cngb.org/) under accession numbers CNR0552654 and CNR0552655, respectively. The sequencing data of C. argyrosperma, C. cordata, C. digitata, C. ecuadorensis, C. foetidissima, C. lundelliana, C. okeechobeensis, and C. pedatifolia are in the NCBI (https://www.ncbi.nlm.nih.gov/) dataset with accession numbers SRR7685402, SRR2531290, SRR26763386, SRR26753257, SRR11573110, SRR26763385, SRR26761657, and SRR26761658, respectively. The chloroplast genome sequences of C. pepo (NC_038229.1) and C. ficifolia (NC_058583.1) were directly downloaded from the NCBI. Fully assembled and annotated plastomes also have been deposited in NCBI and CNGBdb. (C. moschata: N_002031915; C. maxima: N_002031914; C. argyrosperma: OL782153; C. cordata: N_001486273; C. digitata: N_001486272; C. ecuadorensis: N_001486274; C. foetidissima: N_001486275; C. lundelliana: N_001486271; C. okeechobeensis: NC_065149.1; and C. pedatifolia: NC_038229.1).
