# Supplementary material for: Comparative complete chloroplast genome analysis of Cucurbita (Cucurbitaceae) species revealed insights into phylogenetic evolution, adaptive pressure, and lineage diversification
Source: Front Plant Sci. 2026 Mar 27;17:1803791. doi: 10.3389/fpls.2026.1803791 (PMC13066293; doi:10.3389/fpls.2026.1803791)
Supplement: Supplementary file 1 [file DataSheet1.docx]

Supplementary Material

# Supplementary Figures and Tables

## Supplementary Figures


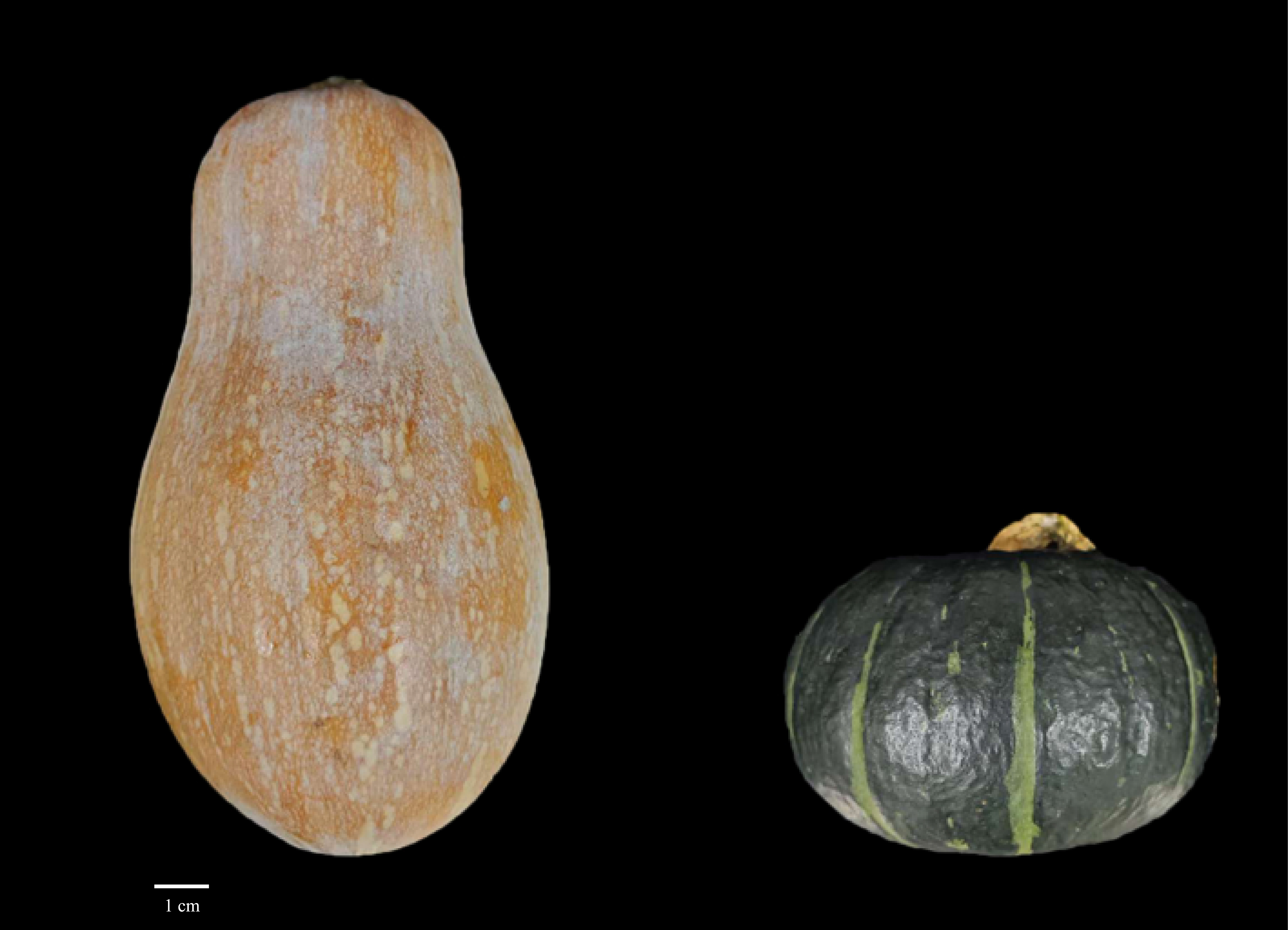


**Supplementary Figure 1.** Two *Cucurbita* species sequenced in this study. Left: *C. moschata*; Right: *C. maxima.*


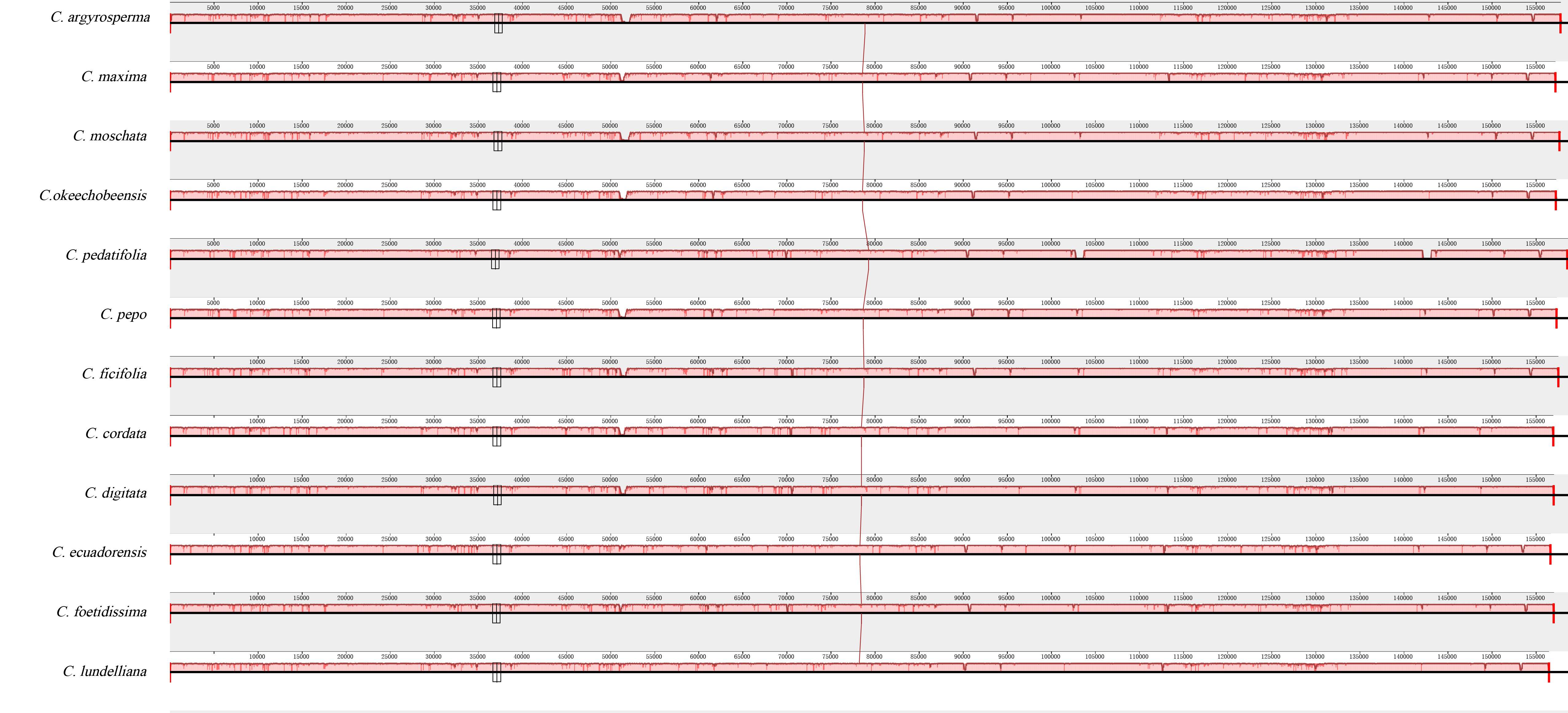


**Supplementary Figure 2.** A collinearity analysis on 12 *Cucurbita* CPGs.





**Supplementary Figure 3.** Cluster analysis of 12 *Cucurbita* species based on the *trnL*-*trnF* region was performed using the neighbor-joining (NJ) method.





**Supplementary Figure 4.** Intraspecific differentiation analysis in *C. moschata* and *C. maxima.* (A) Polymorphism analysis of CPGs from three *C. moschata* individuals, and the 1137bp alignment sequences with Pi≥0.4 were visualized by Jalview. (B) Polymorphism analysis of CPGs from three *C. maxima* individuals. (C) Clustering analysis was performed based on the three CPGs of *C. moschata* and three CPGs of *C. maxima* using the NJ method.


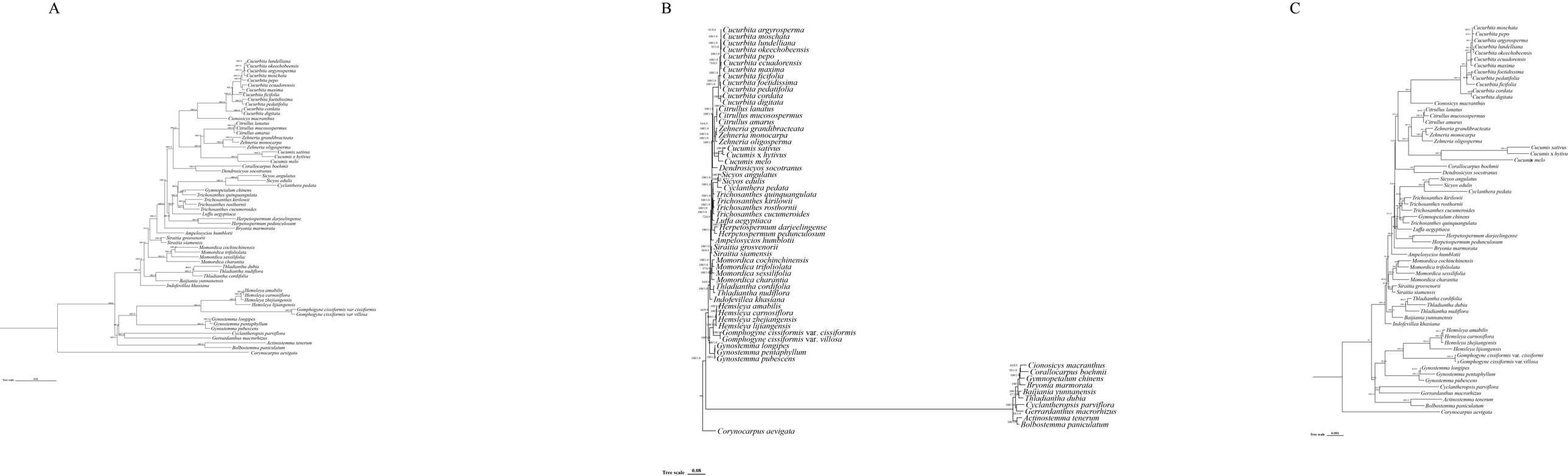


**Supplementary Figure 5.**  ML tree and BI tree showing phylogenetic relationships among 61 Cucurbitaceae species using the LSC region (A), the SSC region (B), and the IRa region (C).

## Supplementary Tables

**Supplementary Table 1** List of species and their basic information included in phylogenetic analyses.

| NO. | Species | Genus | Family | Accession number |
| --- | --- | --- | --- | --- |
| 1 | *Actinostemma tenerum* | *Actinostemma* | Actinostemmateae | PV938953.1 |
| 2 | *Bolbostemma paniculatum* | *Bolbostemma* | Actinostemmateae | NC_072567.1 |
| 3 | *Citrullus amarus* | *Citrullus* | Benincaseae | NC_035974.1 |
| 4 | *Citrullus lanatus* | *Citrullus* | Benincaseae | NC_032008.1 |
| 5 | *Citrullus mucosospermus* | *Citrullus* | Benincaseae | KY430687 |
| 6 | *Cucumis x hytivus* | *Cucumis* | Benincaseae | NC_033871.1 |
| 7 | *Cucumis melo* | *Cucumis* | Benincaseae | MT622320.1 |
| 8 | *Cucumis sativus* | *Cucumis* | Benincaseae | MT721154.1 |
| 9 | *Zehneria grandibracteata* | *Zehneria* | Benincaseae | PP831692.1 |
| 10 | *Zehneria monocarpa* | *Zehneria* | Benincaseae | PP831696.1 |
| 11 | *Zehneria oligosperma* | *Zehneria* | Benincaseae | PP831695.1 |
| 12 | *Bryonia marmorata* | *Bryonia* | Bryonieae | NC_046865.1 |
| 13 | *Corallocarpus boehmii* | *Corallocarpus* | Coniandreae | NC_046874.1 |
| 14 | *Dendrosicyos socotranus* | *Dendrosicyos* | Coniandreae | NC_046862.1 |
| 15 | *Cucurbita lundelliana* | *Cucurbita* | Cucurbiteae | N_001486271 |
| 16 | *Cucurbita ecuadorensis* | *Cucurbita* | Cucurbiteae | N_001486274 |
| 17 | *Cucurbita foetidissima* | *Cucurbita* | Cucurbiteae | N_001486275 |
| 18 | *Cucurbita cordata* | *Cucurbita* | Cucurbiteae | N_001486273 |
| 19 | *Cucurbita digitata* | *Cucurbita* | Cucurbiteae | N_001486272 |
| 20 | *Cucurbita maxima* | *Cucurbita* | Cucurbiteae | NC_036505.1 |
| 21 | *Cucurbita ficifolia* | *Cucurbita* | Cucurbiteae | NC_058583.1 |
| 22 | *Cucurbita okeechobeensis* | *Cucurbita* | Cucurbiteae | NC_065149.1 |
| 23 | *Cucurbita pepo* | *Cucurbita* | Cucurbiteae | NC_038229.1 |
| 24 | *Cucurbita pedatifolia* | *Cucurbita* | Cucurbiteae | NC_065199.1 |
| 25 | *Cucurbita moschata* | *Cucurbita* | Cucurbiteae | NC_036506.1 |
| 26 | *Cucurbita argyrosperma* | *Cucurbita* | Cucurbiteae | NC_065148.1 |
| 27 | *Cionosicys macranthus* | *Cionosicys* | Cucurbiteae | NC_046862.1 |
| 28 | *Indofevillea khasiana* | *Indofevillea* | Indofevilleeae | NC_046859.1 |
| 29 | *Gynostemma longipes* | *Gynostemma* | Gomphogyneae | NC_036140.1 |
| 30 | *Gynostemma pentaphyllum* | *Gynostemma* | Gomphogyneae | KX852298.1 |
| 31 | *Gynostemma pubescens* | *Gynostemma* | Gomphogyneae | NC_036142.1 |
| 32 | *Hemsleya amabilis* | *Hemsleya* | Gomphogyneae | NC_082823.1 |
| 33 | *Hemsleya carnosiflora* | *Hemsleya* | Gomphogyneae | ON880468.1 |
| 34 | *Hemsleya lijiangensis* | *Hemsleya* | Gomphogyneae | NC_039653.1 |
| 35 | *Hemsleya zhejiangensis* | *Hemsleya* | Gomphogyneae | NC_056163.1 |
| 36 | *Gomphogyne cissiformis var. cissiformis* | *Gomphogyne* | Gomphogyneae | MH256801.1 |
| 37 | *Gomphogyne cissiformis var. villosa* | *Gomphogyne* | Gomphogyneae | MF784515.1 |
| 38 | *Ampelosycios humblotii* | *Ampelosycios* | Joliffieae | NC_046869.1 |
| 39 | *Telfairia occidentalis* | *Telfairia* | Joliffieae | NC_067979.1 |
| 40 | *Momordica charantia* | *Momordica* | Momordiceae | NC_036807.1 |
| 41 | *Momordica cochinchinensis* | *Momordica* | Momordiceae | NC_065200.1 |
| 42 | *Momordica sessilifolia* | *Momordica* | Momordiceae | NC_046872.1 |
| 43 | *Momordica trifoliolata* | *Momordica* | Momordiceae | PP646449.1 |
| 44 | *Herpetospermum darjeelingense* | *Herpetospermum* | Schizopeponeae | PQ851716.1 |
| 45 | *Herpetospermum pedunculosum* | *Herpetospermum* | Schizopeponeae | NC_046858.1 |
| 46 | *Gymnopetalum chinens* | *Gymnopetalum* | Sicyoeae | NC_072506.1 |
| 47 | *Sicyos angulatus* | *Sicyos* | Sicyoeae | NC_062884.1 |
| 48 | *Sicyos edulis* | *Sicyos* | Sicyoeae | MN542384.1 |
| 49 | *Cyclanthera pedata* | *Trichosanthes* | Sicyoeae | NC_046860.1 |
| 50 | *Trichosanthes cucumeroides* | *Trichosanthes* | Sicyoeae | NC_080536.1 |
| 51 | *Trichosanthes kirilowii* | *Trichosanthes* | Sicyoeae | PV426762.1 |
| 52 | *Trichosanthes sunhangii* | *Trichosanthes* | Sicyoeae | PQ473692.1 |
| 53 | *Trichosanthes rosthornii* | *Trichosanthes* | Sicyoeae | NC_080912.1 |
| 54 | *Luffa aegyptiaca* | *Trichosanthes* | Sicyoeae | OR327040.1 |
| 55 | *Baijiania yunnanensis* | *Siraitia* | Siraitieae | NC_046871.1 |
| 56 | *Siraitia grosvenorii* | *Siraitia* | Siraitieae | NC_043881.1 |
| 57 | *Siraitia siamensis* | *Siraitia* | Siraitieae | NC_046482.1 |
| 58 | *Thladiantha cordifolia* | *Thladiantha* | Thladiantheae | OR668895.1 |
| 59 | *Thladiantha dubia* | *Thladiantha* | Thladiantheae | NC_046855.1 |
| 60 | *Thladiantha nudiflora* | *Thladiantha* | Thladiantheae | OQ286031.1 |
| 61 | *Cyclantheropsis parviflora* | *Cyclantheropsis* | Triceratieae | NC_046870.1 |

**Supplementary Table 2** The size of total repeats in CPG of 12 *Cucurbita* species.

|  | Total SSR size | CPG size | SSR/CPG |
| --- | --- | --- | --- |
| *C.lundelliana* | 4616 | 156448 | 2.95% |
| *C.ecuadorensis* | 5141 | 156651 | 3.28% |
| *C.foetidissima* | 5303 | 157015 | 3.38% |
| *C.cordata* | 6305 | 156954 | 4.02% |
| *C.digitata* | 6523 | 157039 | 4.15% |
| *C. maxima* | 7472 | 157204 | 4.75% |
| *C.ficifolia* | 12553 | 157533 | 7.97% |
| *C.okeechobeensis* | 14648 | 157273 | 9.31% |
| *C.pepo* | 15082 | 157343 | 9.59% |
| *C.pedatifolia* | 16069 | 158614 | 10.13% |
| *C. moschata* | 19112 | 157644 | 12.12% |
| *C.argyrosperma* | 19651 | 157809 | 12.45% |

**Supplementary Table 3** SSR polymorphism of the chloroplast genome of *Cucurbita*.

| SSR number | Core motif | *C. pepo* | *C.argyrosperma* | *C.okeechobeensis* | *C. pedatifolia* | *C.ficifolia* | *C. maxima* | *C. moschata* | *C. cordata* | *C.foetidissima* | *C.ecuadorensis* | *C.lundelliana* | *C.digitata* |
| --- | --- | --- | --- | --- | --- | --- | --- | --- | --- | --- | --- | --- | --- |
| SSR1 | (T)n | 9 | 15 | 13 | 10 | 10 | 11 | 15 | 10 | 9 | 12 | 13 | 10 |
| SSR3 | (A)n | 11 | 11 | 11 | 11 | 11 | 11 | 11 | 11 | 11 | 11 | 11 | 11 |
| SSR4 | (C)n | 9 | 9 | 10 | 9 | 10 | 8 | 10 | 9 | 9 | 8 | 10 | 9 |
| SSR5 | (A)n | 13 | 11 | 12 | 10 | 15 | 11 | 11 | 12 | 11 | 10 | 12 | 10 |
| SSR7 | (A)n | 11 | 11 | 11 | 12 | 11 | 11 | 11 | 12 | 10 | 11 | 11 | 11 |
| SSR8 | (T)n | 16 | 13 | 12 | 11 | 12 | 11 | 11 | 10 | 10 | 11 | 12 | 10 |
| SSR9 | (A)n | 14 | 13 | 13 | 9 | 11 | 17 | 12 | 9 | 9 | 15 | 13 | 9 |
| SSR10 | (A)n | 13 | 15 | 16 | 16 | 17 | 15 | 16 | 15 | 17 | 15 | 16 | 15 |
| SSR11 | (A)n | 17 | 17 | 17 | 16 | 13 | 14 | 17 | 17 | 15 | 15 | 16 | 17 |
| SSR12 | (A)n | 10 | 9 | 9 | 10 | 10 | 11 | 9 | 14 | 11 | 10 | 9 | 15 |
| SSR14 | (A)n | 11 | 19 | 15 | 9 | 12 | 16 | 16 | 12 | 9 | 16 | 15 | 12 |
| SSR16 | (T)n | 11 | 11 | 11 | 11 | 11 | 10 | 11 | 11 | 10 | 10 | 11 | 10 |
| SSR17 | (T)n | 10 | 9 | 10 | 10 | 10 | 9 | 10 | 11 | 10 | 9 | 10 | 11 |
| SSR18 | (A)n | 10 | 10 | 10 | 14 | 10 | 12 | 9 | 10 | 16 | 11 | 9 | 10 |
| SSR20 | (T)n | 11 | 9 | 9 | 10 | 10 | 12 | 9 | 9 | 9 | 11 | 9 | 9 |
| SSR21 | (T)n | 10 | 10 | 10 | 10 | 10 | 10 | 10 | 10 | 10 | 10 | 10 | 10 |
| SSR23 | (A)n | 14 | 14 | 14 | 15 | 11 | 14 | 15 | 16 | 14 | 15 | 14 | 15 |
| SSR24 | (T)n | 9 | 9 | 9 | 9 | 10 | 5 | 9 | 8 | 9 | 9 | 9 | 9 |
| SSR29 | (T)n | 10 | 9 | 10 | 10 | 11 | 10 | 10 | 9 | 11 | 9 | 10 | 9 |
| SSR30 | (A)n | 12 | 11 | 11 | 10 | 10 | 11 | 11 | 9 | 10 | 10 | 11 | 9 |
| SSR31 | (A)n | 17 | 16 | 17 | 15 | 14 | 18 | 17 | 9 | 14 | 15 | 17 | 9 |
| SSR32 | (A)n | 10 | 11 | 10 | 14 | 11 | 10 | 11 | 10 | 14 | 10 | 10 | 11 |
| SSR34 | (T)n | 9 | 9 | 9 | 10 | 10 | 9 | 10 | 12 | 10 | 9 | 9 | 11 |
| SSR37 | (T)n | 11 | 11 | 11 | 13 | 11 | 9 | 12 | 12 | 13 | 11 | 11 | 11 |
| SSR38 | (T)n | 7 | 7 | 7 | 6 | 10 | 6 | 7 | 6 | 6 | 6 | 7 | 6 |
| SSR40 | (G)n | na | na | na | 10 | 10 | na | na | 8 | 10 | na | na | 8 |
| SSR41 | (A)n | 10 | 10 | 10 | 9 | 12 | 10 | 10 | 11 | 9 | 10 | 10 | 11 |
| SSR42 | (A)n | 9 | 9 | 9 | 15 | 15 | 9 | 9 | 15 | 17 | 9 | 9 | 15 |
| SSR43 | (T)n | 10 | 11 | 12 | 11 | 12 | 12 | 11 | 8 | 11 | 10 | 12 | 11 |
| SSR44 | (T)n | 8 | 9 | 9 | 9 | 10 | 9 | 9 | 10 | 10 | 9 | 9 | 10 |
| SSR45 | (T)n | 13 | 12 | 11 | 11 | 11 | 12 | 12 | 13 | 12 | 12 | 11 | 13 |
| SSR47 | (T)n | 9 | 9 | 9 | 8 | 10 | 9 | 9 | 9 | 8 | 9 | 9 | 10 |
| SSR51 | (AT)n | 4 | 4 | 4 | 4 | 6 | 4 | 4 | 4 | 4 | 4 | 4 | 4 |
| SSR53 | (A)n | 9 | 9 | 9 | 9 | 10 | 9 | 9 | 9 | 9 | 10 | 9 | 9 |
| SSR54 | (AT)n | 5 | 5 | 5 | 5 | 5 | 5 | 6 | 5 | 5 | 5 | 5 | 5 |
| SSR55 | (A)n | 10 | 9 | 8 | 9 | 10 | 11 | 9 | 9 | 9 | 10 | 8 | 9 |
| SSR56 | (T)n | 7 | 10 | 10 | 12 | 11 | 11 | 12 | 10 | 7 | 10 | 10 | 10 |
| SSR57 | (A)n | 9 | 9 | 9 | 9 | 11 | 10 | 8 | 11 | 10 | 9 | 9 | 11 |
| SSR59 | (T)n | 10 | 10 | 10 | 10 | 10 | 10 | 10 | 8 | 10 | 9 | 10 | 8 |
| SSR62 | (T)n | 8 | 8 | 8 | 8 | 10 | 8 | 8 | na | 8 | 8 | 8 | na |
| SSR63 | (TA)n | 4 | 4 | 4 | 4 | 6 | 4 | 4 | 4 | 4 | 4 | 4 | 4 |
| SSR65 | (A)n | 11 | 11 | 12 | 11 | 10 | 11 | 11 | 11 | 12 | 12 | 13 | 10 |

**Supplementary Table 4** SSR polymorphism of the chloroplast genome of *Cucurbita*.

| SSR number | SSR type | SSR | size | start | end | Location | FORWARD PRIMER1 (5'-3') | REVERSE PRIMER1 (5'-3') | PRODUCT1 size (bp) |
| --- | --- | --- | --- | --- | --- | --- | --- | --- | --- |
| 1 | p1 | (T)10 | 10 | 1532 | 1541 | *psbA-trnK* | CCCATGTCAACCAATATCAACA | ATCCGACTAGTTCCGGGTTC | 231 |
| 3 | p1 | (A)10 | 10 | 4960 | 4969 | *matK-rps16* | GCAACAAAATAACCCCATTGA | CCGTCTTCGATAACGACAAAA | 178 |
| 4 | p1 | (C)10 | 10 | 5366 | 5375 | *rps16* | CTCTTCCTTCTCTTCGGGCT | TTGAATTTCCTCGAGCCGTA | 137 |
| 5 | p1 | (A)15 | 15 | 5537 | 5551 | *rps16* | GTACGGCTCGAGGAAATTCA | GGGGTTAGAGACCGCTCAAT | 248 |
| 7 | p1 | (A)11 | 11 | 6707 | 6717 | *rps16-trnQ* | GAATGGATTCACGAGTTTCACA | TTATGGATGGGTTAATCCGAA | 184 |
| 8 | p1 | (T)12 | 12 | 7270 | 7281 | *rps16-trnQ* | TGGACTAGTTTCGGAGGTGC | TGGTGTGTGAAACCCGAGTA | 240 |
| 9 | p1 | (A)11 | 11 | 7398 | 7408 | *rps16-trnQ* | TGGACTAGTTTCGGAGGTGC | TAAATGGTGTGTGAAACCCG | 244 |
| 10 | p1 | (A)17 | 17 | 8073 | 8089 | *trnQ-psbK* | TGAAGGATTTTTGATTGGCTG | GTAGGCCTCAGGCAATTTGG | 267 |
| 11 | p1 | (A)13 | 13 | 8629 | 8641 | *trnQ-psbK* | CCTGTTAGAGTCCCCCACAA | CGTCCCGGGTCATTAGATAG | 280 |
| 12 | p1 | (A)10 | 10 | 9294 | 9303 | *trnS-trnR* | AAAGTAAGGGCTCAAAAGAAGAGA | CAGGCCGTGGAAATAAAATG | 184 |
| 14 | p1 | (A)12 | 12 | 10772 | 10783 | *trnS-trnR* | CGAACAAGAGCGGTGAAAAT | TGCGTCCAATAGGATTTGAA | 119 |
| 16 | p1 | (T)11 | 11 | 14365 | 14375 | *atpF-atpH* | GATGGCCAGTAACCCAAAGA | TGGGAAGGATTGATTTGAGG | 253 |
| 17 | p1 | (T)10 | 10 | 15535 | 15544 | *atpF-atpH* | TCGAAACATCCACAAGGGTT | TGGAAGGATTCGGACTAATGA | 175 |
| 18 | p1 | (A)10 | 10 | 17582 | 17591 | *rps2-rpoC2* | TGCTTCCATCATCTCTTCCA | AACTATCCGCGTTGATTTGG | 237 |
| 20 | p1 | (T)10 | 10 | 24252 | 24261 | *rpoC2-rpoC1* | GGTTATGCCACGATGTCCTT | GACATGTGGCTTGCTTGGTA | 279 |
| 21 | p1 | (T)10 | 10 | 27507 | 27516 | *rpoB-trnC* | GGATCTCCGCCTACACAAGA | TCGAAAAGCAAGGATATGGG | 222 |
| 23 | p1 | (A)11 | 11 | 30445 | 30455 | *petN-psbM* | TGGGGAAGAAGTGGACTCTA | CACGTAAGATGTGGGTGGCT | 278 |
| 24 | p1 | (T)10 | 10 | 31608 | 31617 | *psbM-trnD* | ACCGAATTCAAACAAAACGG | AATTGCATTGGACAAGAGGG | 121 |
| 29 | p1 | (T)11 | 11 | 44514 | 44524 | *psaA-ycf3* | CCTCTTTCCGGACAACACAT | CAATTCAAAACCCGGAAAAA | 247 |
| 30 | p1 | (A)10 | 10 | 45099 | 45108 | *psaA-ycf3* | TGATGCGGACAACAAACAAT | GGTTGAAGATCACAAGGCGT | 223 |
| 31 | p1 | (A)14 | 14 | 46963 | 46976 | *ycf3-trnS* | GCACCTCCAAGAAGCGTAGA | AAATGGGGTTGTTACCGATG | 261 |
| 32 | p1 | (A)11 | 11 | 47666 | 47676 | *ycf3-trnS* | AGAATTGGTTGGACGTGCCT | CGAACCCTCGGTAAACAAAA | 159 |
| 34 | p1 | (T)10 | 10 | 52060 | 52069 | *trnF-ndhJ* | GGCACATCATGAATTTGTATGC | AATTGAACGCGAGGAGACAG | 279 |
| 37 | p1 | (T)11 | 11 | 63332 | 63342 | *psaI-ycf4* | GTAGGCCTAGTATTTCCGGC | CTTATGTTTGGAGTCGGCCA | 180 |
| 38 | p1 | (T)10 | 10 | 68800 | 68809 | *psbE-petL* | TCGGTTATTGAGACAGCGAA | TGCGTGCATTACGAATATTGA | 280 |
| 40 | p1 | (G)10 | 10 | 70588 | 70597 | *trnP-psaJ* | CCGCCGTAAAGAAAAATGAA | CCGCTTGTATTGTACGCTGA | 138 |
| 41 | p1 | (A)12 | 12 | 72846 | 72857 | *rpl20-rps12* | TATAACCTTCCCGACCACGA | GAGCCAAAGAGGGTGAACTG | 248 |
| 42 | p1 | (A)15 | 15 | 74525 | 74539 | *clpP-psbB* | TGAAAGAGGCCTCCGATAAA | ACGTCTAGCATTCCCTCACG | 240 |
| 43 | p1 | (T)12 | 12 | 75290 | 75301 | *clpP-psbB* | TAAAGATCTGCCCGATTTGG | TATCCAGGCTCCGCTTAGAA | 260 |
| 44 | p1 | (T)10 | 10 | 81691 | 81700 | *petD-rpoA* | CGGATTCCGTTAAAGATTCA | AATTGCCTCAAAAGGTCCAA | 228 |
| 45 | p1 | (T)11 | 11 | 83891 | 83901 | *infA-rps8* | TCATTCCAGATGAAACCCGT | GACAGACCGAGAAGCTCGAC | 179 |
| 47 | p1 | (T)10 | 10 | 88133 | 88142 | *rps19-rpl2* | TGGTAGATGCTCTGGACCAA | GGGTTATCCTGCACTTGGAA | 269 |
| 51 | p2 | (AT)6 | 12 | 112710 | 112721 | *trnN-ndhF* | CCATCCATATCCCAATTCCA | ACCACAACGACCGAATTGAT | 246 |
| 53 | p1 | (A)10 | 10 | 118962 | 118971 | *ccsA-ndhD* | TGGTTCTCACAAAACCCAAA | TCAGTTGACAAGGTCGAAGC | 205 |
| 54 | p2 | (AT)5 | 10 | 120578 | 120587 | *ccsA-ndhD* | CCCGCAAATATTGGAAAAAC | GCATGAAACAACTCGAAGCA | 237 |
| 55 | p1 | (A)10 | 10 | 123948 | 123957 | *ndhA-ndhH* | GCCCCCAATTAACCCAATAG | TTCGTAAGTGAAAACGATGGG | 173 |
| 56 | p1 | (T)11 | 11 | 124192 | 124202 | *ndhA-ndhH* | GTTCATGAATTCGGACACGA | ATTCCGATCCAGAGTATGCG | 249 |
| 57 | p1 | (A)11 | 11 | 124793 | 124803 | *ndhA-ndhH* | TAAAGGCTCTCTTGCGCCTA | ACGTGTGATTCGTTGAGACA | 234 |
| 59 | p1 | (T)10 | 10 | 128549 | 128558 | *ycf1-trnN* | TCCAGATTCGATATGGAACGA | AGGCCGATATTAGAGCCTGG | 232 |
| 62 | p1 | (T)10 | 10 | 131020 | 131029 | *ycf1-trnN* | CGAAAATCCGATAGTTGGGA | GTTAGAACGACGGGAGCAAG | 258 |
| 63 | p2 | (TA)6 | 12 | 132925 | 132936 | *ycf1-trnN* | ACCACAACGACCGAATTGAT | CCATCCATATCCCAATTCCA | 246 |
| 65 | p1 | (A)10 | 10 | 143111 | 143120 | *ycf15-rps7* | CGTACCTTTCGCTCAATGAA | TCATTGGATCCTTTTCCTCG | 243 |

**Supplementary Table 5** Statistics and comparison of published CPGs in *Cucurbita* and the genomes assembled in this study

| **Species** | **Accession number** | **Size** | **Number of gaps** | **Number of genes** | **Reference** |
| --- | --- | --- | --- | --- | --- |
| *C. ficifolia* | NC_058583.1/MZ578000.1 | 157,533 bp | 0 | 131 | Zhang et al., 2021 |
|  | MW801448 | 157,631 bp | 0 | 128 | He et al., 2021 |
|  | KT898809 | 156,711 bp | 2 | 103 | Kistler et al., 2015 |
| *C. pepo* | NC_038229.1/MH031787.1 | 157,343 bp | 0 | 131 | Zhou et al., 2018 |
|  | KT898819 | 156,541 bp | 5 | 103 | Kistler et al., 2015 |
| *C. moschata* | N_002031915 | 157,564 bp | 0 | 131 | —— |
|  | OQ442842.1 | 157,592 bp | 0 | 136 | available in NCBI |
|  | NC_036506.1 | 157,644 bp | 0 | 135 | available in NCBI |
|  | KT898813 | 156,724 bp | 2 | 103 | Kistler et al., 2015 |
| *C. maxima* | N_002031914 | 157,461 bp | 0 | 131 | —— |
|  | NC_036505.1 | 157,204 bp | 0 | 130 | available in NCBI |
|  | OK129338.1 | 157,205 bp | 0 | 132 | available in NCBI |
| *C. okeechobeensis* | NC_065149.1/OL782154.1 | 157,273 bp | 0 | 131 | —— |
|  | KT898814 | 156,611 bp | 2 | 103 | Kistler et al., 2015 |
| *C. pedatifolia* | NC_065199.1/ON597625.1 | 158614 bp | 0 | 131 | —— |
|  | KT898821 | 156,544 bp | 2 | 102 | Kistler et al., 2015 |
| *C. argyrosperma* | NC_065148.1/OL782153.1 | 157,809 bp | 0 | 131 | —— |
|  | KT898803 | 156,757 bp | 2 | 104 | Kistler et al., 2015 |
|  | CM014103.1 | 157,623 bp | 0 | unkown | Barrera-Redondo et al., 2019 |
| *C. ecuadorensis* | N_001486274 | 156,651 bp | 0 | 131 | —— |
|  | KT898808 | 156,664 bp | 6 | 102 | Kistler et al., 2015 |
| *C. foetidissima* | N_001486275 | 157015 bp | 0 | 131 | —— |
|  | KT898810 | 156,776 bp | 6 | 102 | Kistler et al., 2015 |
| *C. cordata* | N_001486273 | 156,954 bp | 0 | 131 | —— |
|  | KT898806 | 156,759 bp | 2 | 101 | Kistler et al., 2015 |
| *C. digitata* | N_001486272 | 157039 bp | 0 | 131 | —— |
|  | KT898807 | 156,835 bp | 4 | 101 | Kistler et al., 2015 |

note:"—" indicates samples gained from this study.
